# Preferred lifestyle intervention characteristics and behaviour change needs of postpartum women following cardiometabolic pregnancy complications

**DOI:** 10.1177/17455057241247748

**Published:** 2024-07-26

**Authors:** Elaine K Osei-Safo, Siew Lim, Maureen Makama, Mingling Chen, Helen Skouteris, Frances Taylor, Cheryce L Harrison, Melinda Hutchesson, Christie J Bennett, Helena Teede, Angela Melder, Lisa J Moran

**Affiliations:** 1 Monash Centre for Health Research and Implementation (MCHRI), Monash University, Clayton, VIC, Australia; 2 Eastern Health Clinical School, Monash University, Box Hill, VIC, Australia; 3 Health and Social Care Unit, Monash University, Clayton, VIC, Australia; 4 Warwick Business School, Coventry, UK; 5 The University of Newcastle, Callaghan, NSW, Australia; 6 Department of Nutrition, Dietetics and Food, Monash University, Notting Hill, VIC, Australia

**Keywords:** adverse pregnancy outcome, cardiometabolic disease, cardiometabolic pregnancy complication, gestational diabetes mellitus, hypertensive disorders of pregnancy, intrauterine growth restriction, lifestyle intervention, postpartum, spontaneous preterm birth, women’s health

## Abstract

**Background:**

Women with cardiometabolic pregnancy complications are at increased risk of future diabetes and heart disease which can be reduced through lifestyle management postpartum.

**Objectives:**

This study aimed to explore preferred intervention characteristics and behaviour change needs of women with or without prior cardiometabolic pregnancy complications for engaging in postpartum lifestyle interventions.

**Design:**

Quantitative cross-sectional study.

**Methods:**

Online survey.

**Results:**

Overall, 473 women were included, 207 (gestational diabetes (n = 105), gestational hypertension (n = 39), preeclampsia (n = 35), preterm birth (n = 65) and small for gestational age (n = 23)) with and 266 without prior cardiometabolic pregnancy complications. Women with and without complications had similar intervention preferences, with delivery ideally by a healthcare professional with expertise in women’s health, occurring during maternal child health nurse visits or online, commencing 7 weeks to 3 months post birth, with 15- to 30-min monthly sessions, lasting 1 year and including monitoring of progress and social support. Women with prior complications preferred intervention content on women’s health, mental health, exercise, mother’s diet and their children’s health and needed to know more about how to change behaviour, have more time to do it and feel they want to do it enough to participate. There were significant differences between groups, with more women with prior cardiometabolic pregnancy complications wanting content on women’s health (87.9% vs 80.8%, p = 0.037), mother’s diet (72.5% vs 60.5%, p = 0.007), preventing diabetes or heart disease (43.5% vs 27.4%, p < 0.001) and exercise after birth (78.3% vs 68.0%, p = 0.014), having someone to monitor their progress (69.6% vs 58.6%, p = 0.014), needing the necessary materials (47.3% vs 37.6%, p = 0.033), triggers to prompt them (44.0% vs 31.6%, p = 0.006) and feeling they want to do it enough (73.4%, 63.2%, p = 0.018).

**Conclusion:**

These unique preferences should be considered in future postpartum lifestyle interventions to enhance engagement, improve health and reduce risk of future cardiometabolic disease in these high-risk women.

## Introduction

Cardiometabolic pregnancy complications can be defined as maternal and foetal complications experienced during gestation that can contribute to an increased risk of maternal type 2 diabetes (T2D) and cardiovascular disease (CVD) post birth.^1^ Their pathophysiology is underpinned by various factors, including impaired hemodynamic adaptation, endothelial and cardiac dysfunction, placental insufficiency, inflammation, oxidative stress, insulin resistance and alterations in glucose metabolism.^1–3^ Cardiometabolic pregnancy complications include gestational diabetes mellitus (GDM), hypertensive disorders of pregnancy including preeclampsia (PE), alongside some causes of spontaneous preterm birth (PTB), intrauterine growth restriction (IUGR) and giving birth to a small for gestational age (SGA) infant. Together these affect up to 30% of singleton pregnancies.^1,4^ Modifiable risk factors for these conditions include high blood pressure, high cholesterol, dyslipidemia, insulin resistance and glucose intolerance, a higher weight, physical inactivity, inadequate nutrition, cigarette smoking and stress.^1,2,4–6^

Pregnancy is often referred to as a natural cardiac stress test, as it may unmask underlying risks of suboptimal cardiovascular health.^5^ Experiencing a cardiometabolic pregnancy complication presents a sex-specific risk factor for future cardiometabolic disease development^6^ and is associated with up to a 10-fold and 2-fold increased risk of T2D^7^ and CVD, respectively, in the postpartum period and beyond.^8,9^ For these women, the postpartum period presents a unique opportunity to provide healthy lifestyle interventions to reduce future cardiometabolic risk.

Current guidelines for managing cardiometabolic pregnancy complications postpartum suggest providing patient-centred, culturally sensitive and practical lifestyle counselling on optimizing diet, exercise and weight and engaging in regular cardiometabolic screening.^10,11^ However, the postpartum period presents a challenging life stage for many women, representing a time of substantial physical and emotional changes, competing demands and new responsibilities.^12–18^ Barriers to adopting a healthy lifestyle for all women postpartum may include lack of knowledge regarding the benefits of lifestyle behaviours, low-risk perception of future lifestyle-related diseases and lack of motivation, time, energy, resources, social support, and individualized and culturally sensitive health advice from healthcare professionals.^12–19^ Furthermore, some healthcare professionals may feel they require more training to provide appropriate postpartum lifestyle support.^12^ At a system level, there is also a lack of funding for lifestyle interventions.^20^ These barriers may contribute to the low levels of intervention uptake in postpartum lifestyle interventions.^21^ Our prior systematic review noted participation rates of 0.94%–86% in postpartum lifestyle interventions; however, we acknowledge the limited number of studies included with high-risk populations (4/36), indicating further work is needed in understanding how to support these women.^21^

Lifestyle interventions are more effective when using evidence-based theoretical frameworks in design and implementation.^22^ For example, the Capability, Opportunity, Motivation and Behaviour (COM-B) system identifies capability, opportunity and motivation as essential factors facilitating behaviour change.^23^ The Template for Intervention Description and Replication (TIDieR) checklist provides a comprehensive guide for describing intervention characteristics (e.g. why, what, who, how, where, when and how much) to aid in improving intervention efficacy and replicability.^24^ These frameworks are used frequently to inform successful postpartum intervention design^15,25^ with the TIDieR checklist previously used in identifying intervention characteristics associated with greater postpartum weight loss^25^ and the COM-B system in identifying barriers and enablers experienced by women from culturally diverse backgrounds with prior GDM.^15^ However, these studies focused on the general postpartum population or women with prior GDM. It is therefore crucial to explore the perspectives and needs of postpartum women with a range of cardiometabolic pregnancy complications to optimize lifestyle intervention content and delivery and improve intervention relevance, uptake and effectiveness.

For women who have experienced a cardiometabolic pregnancy complication, understanding what influences behaviour change and preferred intervention characteristics enables development of patient-centred interventions tailored to their wants, needs and preferences. This may consequently increase uptake, engagement, sustainability and behaviour change. Comparing these preferences to postpartum women without prior complications will help identify how these high-risk women could be cared for differently regarding intervention design and delivery. The aims of the study were to explore and compare the interest in, preferred intervention characteristics (based on the TIDieR checklist) and behaviour change needs (based on the COM-B system) of women with or without prior cardiometabolic pregnancy complications for engagement in a postpartum lifestyle intervention.

## Materials and methods

### Study design

This is a sub-study of a quantitative cross-sectional online survey previously conducted to inform the engagement of postpartum women in lifestyle management.^26^ It was approved by the Monash University Human Research Ethics Committee (HREC; Project no. 29273), and all participants provided written informed consent.^26^ Detailed methods are previously described elsewhere.^26^ The STROBE guideline for cross-sectional studies was followed when preparing the article.

### Study participants

Participants were recruited via an external cross-panel market research provider (Octopus group) between 8 November and 21 November 2021.^26^ Eligible participants were women aged 18 years and older who had and had not experienced a cardiometabolic pregnancy complication and delivered their baby in the last 5 years, were not pregnant and were living with their child in Australia. Ineligible participants were women below 18 years of age, who had not delivered a baby within the last 5 years, who were pregnant or were not living with their child in Australia. Women were also excluded who had experienced another health complication which may affect their lifestyle habits or risk of T2D or CVD, specifically diabetes, polycystic ovary syndrome, infertility or experiencing menopause. Participants were generally a broad representation of the Australian population by location and residence in accordance with the Australian Bureau of Statistics.^26^

### Data collection

The survey was self-administered, 20–30 min in duration, consisting of both open format and multiple-choice questions with and without a Likert-type scale response. For this sub-study, the survey questions analysed comprised of a range of questions on the following topics: demographic characteristics (history of cardiometabolic pregnancy complication, age, body mass index (BMI), age of the youngest child, cultural/ethnic background, country of birth, time since migration to Australia (if overseas born), marital status, education, employment, income) and dissemination mode to receive information about lifestyle management (preferred avenue for learning about the programme). Intervention characteristic preferences (according to the TIDieR checklist; preferred programme provider, content, additional inclusions, setting, delivery mode, session frequency, session duration, programme duration) and behaviour change needs (according to the COM-B system; capability, opportunity, motivation; prefaced by: ‘When it comes to you personally participating in a health and wellbeing programme for women after childbirth, what do you think it would take for you to participate in the programme? I would have to. . .’).^26^ The survey was developed by the research team. Survey questions relevant to the COM-B system were adapted from the COM-B Self-Evaluation Questionnaire Volume 1.^27^ The survey was pilot tested on four women and revised as required before the commencement of data collection.^26^

### Statistical analysis

All analyses were performed using SPSS Statistics 28 (IBM Australia Limited, New South Wales, Australia, 2021). Descriptive statistics were used to summarize participant characteristics, dissemination mode, intervention characteristic preferences and behaviour change needs from quantitative data. Categorical data were reported as frequencies and percentages, and continuous data as means and standard deviations for normally distributed data and medians and interquartile ranges for non-normally distributed data. Differences in participant characteristics, choice of dissemination mode, intervention characteristic preferences and behaviour change needs were assessed using an independent sample t-test, Mann–Whitney U test and Pearson’s chi-square test as appropriate, with the significance level set to 0.05, for women with and without a cardiometabolic pregnancy complication. Due to the small sample size of women with cardiometabolic pregnancy complications other than GDM (gestational hypertension; n = 39, PE; n = 35, PTB; n = 65, SGA; n = 23), it was not possible to separately analyse these subgroups. Instead, comparisons were performed by a three-way chi-square test between women who experienced GDM +/− another cardiometabolic pregnancy complication (gestational hypertension, PE, PTB and SGA; n = 105), women who experienced a cardiometabolic pregnancy complication (gestational hypertension, PE, PTB and SGA) without GDM (n = 102) and women who did not experience a cardiometabolic pregnancy complication (n = 266). These groups were chosen as there are more established guidelines for pregnancy and postpartum management of GDM.^28^ Where there was a significant difference between the three groups, a post hoc test with significance level set to 0.016 (due to three pairwise comparisons being performed, i.e. 0.05/3) was conducted to determine between which of these three groups there was a significance difference.

## Results

### Participant characteristics

Of the 473 women, 207 experienced a cardiometabolic pregnancy complication (50.7% GDM, 18.8% gestational hypertension, 16.9% PE, 31.4% PTB and 11.1% had given birth to a SGA infant) and 266 reported having a healthy pregnancy. Of the women who had experienced a cardiometabolic pregnancy complication, 157 (75.8%) experienced one, 50 (24.2%) more than one, 42 (20.3%) more than two, 6 (2.9%) more than three and 2 (1.0%) more than four cardiometabolic pregnancy complications. However, 105 (50.7%) women who experienced a cardiometabolic pregnancy complication experienced GDM +/− another cardiometabolic pregnancy complication and the remaining 102 (49.2%) women experienced a cardiometabolic pregnancy complication without GDM. Participant characteristics are provided in Table 1. Women with a cardiometabolic pregnancy complication had a higher BMI (kg/m^2^) (27.0 ± 10.4 vs 24.9 ± 8.3, p < 0.001) and were more likely to have a BMI ⩾ 25 kg/m^2^ (58.9% vs 48.5%, p = 0.022). There was a significant difference in the age of the youngest child (p = 0.009), country of birth (p = 0.011) and employment status (p = 0.034) between the two groups.

**Table 1.** Characteristics of women who experienced or did not experience a cardiometabolic pregnancy complication.

Characteristic	CMPC (n = 207)	No CMPC (n = 266)	p
Cardiometabolic pregnancy complication			NA
Gestational diabetes mellitus	105 (50.7)	NA	
Gestational hypertension	39 (18.8)	NA	
Preeclampsia	35 (16.9)	NA	
Preterm birth	65 (31.4)	NA	
Small for gestational age	23 (11.1)	NA	
Age (years) (M ± SD)	33.9 ± 5.9	33.2 ± 5.3	0.158
BMI (kg/m^2^) (median ± IQR)	27.0 ± 10.4	24.9 ± 8.3	<0.001
BMI ⩾ 25 kg/m^2^	122 (58.9)	129 (48.5)	0.022
Age of the youngest child			0.009
Less than 6 months	20 (9.7)	38 (14.3)	
6 months to less than 1 year	29 (14.0)	36 (13.5)	
1 year old	46 (22.2)	35 (13.2)	
2 years old	42 (20.3)	44 (16.5)	
3 years old	27 (13.0)	39 (14.7)	
4 years old	26 (12.6)	34 (12.8)	
5 years old	17 (8.2)	40 (15.0)	
Cultural or ethnic background			0.061
Oceanian	177 (85.5)	126 (47.4)	
European	5 (2.4)	13 (4.9)	
African	10 (4.8)	7 (2.6)	
Asian	60 (29.0)	95 (35.7)	
North American	1 (0.5)	7 (2.6)	
Latin American	4 (2.0)	9 (3.4)	
Other	7 (3.4)	5 (1.9)	
Country of birth			0.011
Australian born	123 (59.4)	132 (49.6)	
Overseas born	84 (40.6)	134 (50.4)	
Time since migration to Australia (overseas born), years			0.937
⩽5	24 (28.6)	43 (32.1)	
6–10	28 (33.3)	48 (35.8)	
11–15	14 (16.7)	20 (14.9)	
⩾16	18 (21.4)	23 (17.2)	
Marital status			0.050
Married/de facto	176 (85.0)	239 (89.8)	
Single (never married/divorced/separated)	31 (15.0)	24 (9.0)	
Widowed	0 (0)	0 (0)	
Education			0.532
Primary/elementary school or less	0 (0)	0 (0)	
Secondary/high school	56 (27.1)	64 (24.1)	
Diploma/advanced diploma	43 (20.8)	49 (18.4)	
Degree/higher	108 (52.2)	152 (57.1)	
Employment			0.034
Unemployed/homemaker	66 (31.9)	62 (23.3)	
Employed (full-time/part-time/casual)	130 (62.8)	148 (55.6)	
Studying	2 (1.0)	11 (4.1)	
Retired	0 (0)	0 (0)	
Government assistance	6(2.9)	5 (1.9)	
Income			0.732
Low (<AUD$50,000)	34 (16.4)	42 (15.8)	
Medium (AUD$50,000–AUD$124,999)	99 (47.8)	125 (47.0)	
High (⩾AUD$125,000)	59 (28.5)	87 (32.7)	

CMPC: cardiometabolic pregnancy complication; NA: not applicable; SD: standard deviation; BMI: body mass index; IQR: interquartile range.

Data are presented as n (%), M ± SD or median ± IQR. Data were analysed using independent samples t-test, Mann–Whitney U test and Pearson’s chi-square test as appropriate.

### Dissemination mode to receive information about lifestyle management and intervention characteristic preferences according to the TIDieR checklist

**Figure 1.**
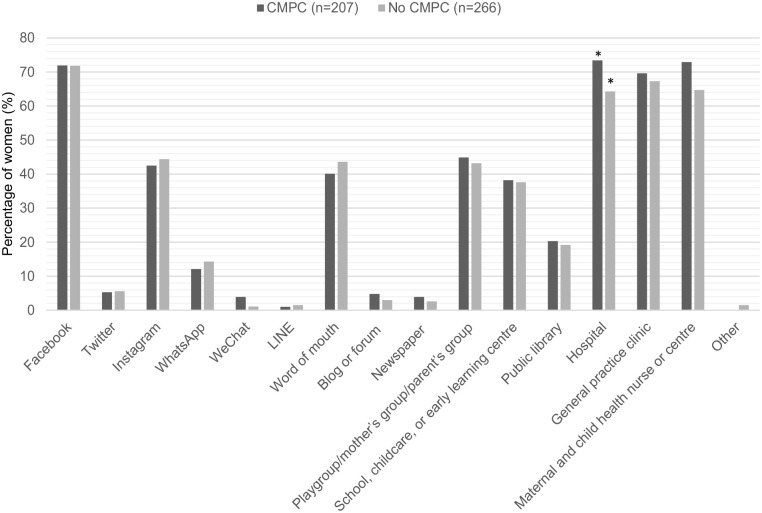
Preferred avenues of women who had or had not experienced a cardiometabolic pregnancy complication for learning about a postpartum lifestyle intervention. Data are presented as percentages (%). Data were analysed using Pearson’s chi-square test. CMPC: cardiometabolic pregnancy complication. *Represents a significant difference between groups.

Both women who had and had not experienced a cardiometabolic pregnancy complication reported being interested in engaging in a postpartum lifestyle intervention (92.8% vs 89.8%, p = 0.297). For women who had experienced a cardiometabolic pregnancy complication, the most preferred avenues for learning about an intervention were hospital (73.4%), maternal child health nurse or centre (72.9%) and Facebook (71.9%). Compared to Facebook (71.8%), general practice clinic (67.3%) and maternal child health nurse or centre (64.7%) for those who did not (Figure 1). There was a significant difference between women who had and had not experienced a cardiometabolic pregnancy complication in hospital as an avenue for learning about a postpartum lifestyle intervention (73.4% vs 64.2%, p = 0.034) (Figure 1).

**Figure 2.**
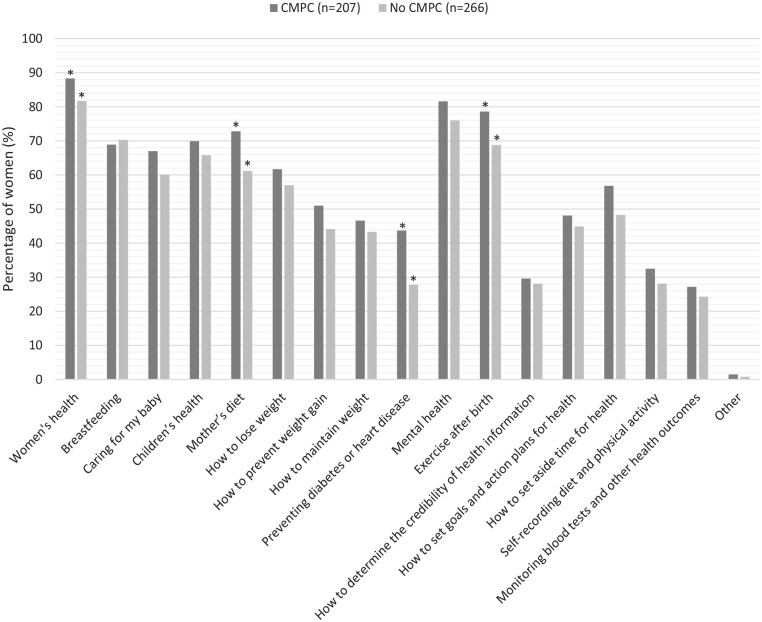
Preferred postpartum lifestyle intervention content of women who had or had not experienced a cardiometabolic pregnancy complication. Data are presented as percentages (%). Data were analysed using Pearson’s chi-square test. CMPC: cardiometabolic pregnancy complication. *Represents a significant difference between groups.

Figure 2 presents the preferred intervention content topics of women. There was a significant difference between women who had and had not experienced a cardiometabolic pregnancy complication in their choice of the topics; women’s health (87.9% vs 80.8%, p = 0.037), mother’s diet (72.5% vs 60.5%, p = 0.007), preventing diabetes or heart disease (43.5% vs 27.4%, p < 0.001) and exercise after birth (78.3% vs 68.0%, p = 0.014). Of those women who had experienced a cardiometabolic pregnancy complication, their five topmost preferences for intervention content were women’s health (87.9%), mental health (81.2%), exercise after birth (78.3%), mother’s diet (72.5%) and their children’s health (69.6%). In comparison, for those who had not experienced a cardiometabolic pregnancy complication, their five topmost preferences for content were women’s health (80.8%), mental health (75.2%), breastfeeding (69.5%), exercise after birth (68.0%) and their children’s health (65.0%).

There was a significant difference between women who experienced GDM +/− another cardiometabolic pregnancy complication, women who experienced a cardiometabolic pregnancy complication without GDM and women who did not experience a cardiometabolic pregnancy complication on intervention content; mother’s diet (78.1% vs 66.7% vs 60.5%, p = 0.006), preventing diabetes or heart disease (51.4% vs 35.3% vs 27.4%, p < 0.001) and exercise after birth (81.9% vs 74.5% vs 68.0%, p = 0.023). A significant post hoc difference was observed between women who had experienced GDM +/− another cardiometabolic pregnancy complication and women who did not experience a cardiometabolic pregnancy complication for all three topics (p = 0.001, p < 0.001, p = 0.007).

Table 2 presents the preferred intervention characteristics of women. There was no difference in preferred intervention provider for women who had or had not experienced a cardiometabolic pregnancy complication, with the top option being someone with expertise in women’s health (e.g. a health professional) for both groups (92.3% vs 89.8%). Both women who had and had not experienced a cardiometabolic pregnancy complication would like additional content inclusions to primarily be; someone to monitor their progress (69.6% vs 58.6%), which was significantly different (p = 0.014), social support for health (67.1% vs 64.3%), for the intervention setting to be during a maternal child health nurse visit (76.8% vs 74.8%) or online (67.6% vs 67.7%) and to be delivered via online information and resources (76.8% vs 72.2%). Women who had and had not experienced a cardiometabolic pregnancy complication generally had similar preferences regarding intervention commencement date (7 weeks to 3 months post birth (40.6% vs 41.0%)), session duration (15–30 min (43.0% vs 44.7%)), session frequency (monthly (35.7% vs 37.2%)) and programme duration (1 year (49.3% vs 43.6%)). There was a significant difference between women who had and had not experienced a cardiometabolic pregnancy complication in their choice of the following intervention frequency; every 3 months (22.7% vs 14.7%, p = 0.024). There was no significant differences between women who had experienced GDM +/− another cardiometabolic pregnancy complication, women who had experienced a cardiometabolic pregnancy complication without GDM and women who did not experience a cardiometabolic pregnancy complication for these intervention characteristics.

**Table 2.** Intervention characteristic preferences of women who had or had not experienced a cardiometabolic pregnancy complication according to the TIDieR checklist.

Intervention characteristic	Responses	CMPC (n = 207)	No CMPC (n = 266)	p
Who: preferred programme provider	Someone with expertise in women’s health (e.g. healthcare professional)	191 (92.3)	239 (89.8)	0.364
	Someone with expertise in children’s health (e.g. healthcare professional)	109 (52.7)	146 (54.9)	0.629
	Another mother	63 (30.4)	64 (24.1)	0.121
	Someone else	4 (1.9)	5 (1.9)	0.967
What: additional inclusions	Someone to monitor my progress	144 (69.6)	156 (58.6)	0.014
	Send me reminders and prompts	130 (62.8)	149 (56.0)	0.516
	Social support for health	139 (67.1)	171 (64.3)	0.194
	Questions to ask my doctor	94 (45.4)	105 (39.5)	0.194
	Other	1 (0.5)	3 (1.1)	0.447
When: preferred time to start	6 weeks or earlier	63 (30.4)	88 (33.1)	0.540
	7 weeks to 3 months	84 (40.6)	109 (41.0)	0.930
	4–6 months	42 (20.3)	50 (18.8)	0.684
	7–12 months	5 (2.4)	10 (3.8)	0.408
	After 12 months	5 (2.4)	2 (0.8)	0.137
	Other	6 (2.9)	4 (1.5)	0.296
Where: preferred setting	Online	140 (67.6)	180 (67.7)	0.993
	Maternal child health nurse visit	159 (76.8)	199 (74.8)	0.615
	Mother’s group/playgroup	108 (52.2)	146 (54.9)	0.557
	GP clinic	117 (56.5)	127 (47.7)	0.058
	Other	4 (1.9)	3 (1.1)	0.472
How: preferred delivery mode	Online information and resource	159 (76.8)	192 (72.2)	0.253
	Print information and resource	76 (36.7)	96 (36.1)	0.889
	One-on-one video or phone consultation	92 (44.4)	109 (41.0)	0.449
	One-on-one face-to-face consultation	115 (55.6)	146 (54.9)	0.885
	Group video consultation	54 (26.1)	57 (21.4)	0.236
	Group face-to-face consultation	83 (40.1)	97 (36.5)	0.420
	Other	0 (0)	1 (0.4)	0.377
How often: preferred session frequency	Every 6 months	8 (3.9)	14 (5.3)	0.474
	Every 3 months	47 (22.7)	39 (14.7)	0.024
	Every month	74 (35.7)	99 (37.2)	0.742
	Every fortnight	44 (21.3)	68 (25.6)	0.274
	Every week	28 (13.5)	37 (13.9)	0.904
	Once off	1 (0.5)	2 (0.8)	0.715
	Other	3 (1.4)	4 (1.5)	0.961
How long: preferred session duration (min)	<15	15 (7.2)	13 (4.9)	0.281
	15–30	89 (43.0)	119 (44.7)	0.705
	30–45	75 (36.2)	92 (34.6)	0.710
	45–60	25 (12.1)	36 (13.5)	0.639
	>60	1 (0.5)	2 (0.8)	0.715
	Other	0 (0)	1 (0.4)	0.277
How long: preferred programme duration	<1 month	8 (3.9)	8 (3.0)	0.609
	1 month	16 (7.7)	29 (10.9)	0.243
	3 months	23 (11.1)	43 (16.2)	0.116
	6 months	53 (25.6)	63 (23.7)	0.630
	1 year	102 (49.3)	116 (43.6)	0.220
	Other	3 (1.4)	4 (1.5)	0.961

CMPC: cardiometabolic pregnancy complication.

Data are presented as n (%) and were analysed using Pearson’s chi-square test.

### Behaviour change needs according to the COM-B system

#### Capability

There was no difference in reported capability between women with and without cardiometabolic pregnancy complications (Table 3). The three main things women with prior cardiometabolic pregnancy complications suggested they would need to participate in a postpartum lifestyle intervention were to know more about how to do it (e.g. have a better understanding of effective ways to increase exercise; 69.1%), know how to create restful time or space for themselves (65.2%) and have more mental strength (e.g. learn how to resist cravings more; 65.2%). In comparison, women without prior cardiometabolic pregnancy complications suggested they would similarly have to have more mental strength (62.4%), know how to create restful time or space for themselves (59.4%) and know more about why it was important (e.g. have a better understanding of how foods affect my health; 58.6%). There was a significant difference between women who experienced GDM +/− another cardiometabolic pregnancy complication, women who experienced a cardiometabolic pregnancy complication without GDM and women who did not experience a cardiometabolic pregnancy complication regarding the following capability needs; known how to organize, plan and prioritize (68.6% vs 57.8% vs 54.5%, p = 0.046). A significant post hoc difference was observed between women who had experienced GDM +/− another cardiometabolic pregnancy complication and women who did not experience a cardiometabolic pregnancy complication for this capability factor (p = 0.013).

**Table 3.** Behaviour change needs of women who have and have not experienced a cardiometabolic pregnancy complication according to the COM-B system.

Behaviour change need	Answer	CMPC (n = 207)	No CMPC (n = 266)	p
Capability: physical and psychological – I would have to. . .	Know more about why it was important (e.g. have a better understanding of how foods affect my health)	129 (62.3)	156 (58.6)	0.418
	Know where to find information	134 (64.7)	150 (56.4)	0.066
	Know more about how to do it (e.g. have a better understanding of effective ways to increase exercise)	143 (69.1)	154 (57.9)	0.130
	Know how to create restful time or space for myself	135 (65.2)	158 (59.4)	0.196
	Have better physical skills (e.g. learn how to cook healthy meals for the family)	89 (43.0)	116 (43.6)	0.894
	Know how to organize, plan and prioritize (e.g. exercise during child’s nap time; incorporate into usual routine, such as taking baby for a walk)	131 (63.3)	145 (54.5)	0.055
	Have more physical strength (e.g. having the fitness to exercise)	131 (63.3)	148 (55.6)	0.094
	Have more mental strength (e.g. learn how to resist cravings more)	135 (65.2)	166 (62.4)	0.528
	Overcome physical limitations (e.g. recovery from childbirth, coping with lack of sleep)	128 (61.8)	155 (58.3)	0.433
	Overcome mental obstacles (e.g. managing stress or negative thoughts about self)	126 (60.9)	149 (56.0)	0.288
	Have more physical stamina (e.g. be able to exercise for longer)	100 (48.3)	112 (42.1)	0.178
	Have more mental stamina (e.g. be able to stick to a plan to eat healthy)	105 (50.7)	135 (50.8)	0.995
	Something else	2 (1.0)	4 (1.5)	0.604
Opportunity: physical and social – I would have to. . .	Have more time to do it (e.g. create a specific time during the day to exercise)	151 (72.9)	192 (72.2)	0.853
	Have a flexible work arrangement (e.g. part-time employment)	86 (41.5)	125 (47.0)	0.237
	Have enough money to do it (e.g. earn enough to pay for gym membership)	118 (57.0)	159 (69.8)	0.544
	Have the necessary materials (e.g. exercise equipment)	98 (47.3)	100 (37.6)	0.033
	Have it more easily accessible (e.g. online access to the intervention)	99 (47.8)	127 (47.7)	0.986
	Have it incorporated with my baby’s appointment (e.g. maternal and child health visits)	95 (45.9)	120 (45.1)	0.866
	Have a conducive environment to do it (e.g. access to recreational facilities and parks)	83 (40.1)	94 (35.3)	0.289
	Have more people around me doing it (e.g. be part of a ‘crowd’ who are doing it)	84 (40.6)	97 (36.5)	0.361
	Have more triggers to prompt me (e.g. have more reminders to exercise at a specific time)	91 (44.0)	84 (31.6)	0.006
	Have the support of my partner on health issues (e.g. verbal and emotional engagement)	91 (44.0)	108 (40.6)	0.463
	Have practical support from others (e.g. help with childcare and chores from partner, family and friends)	120 (58.0)	141 (53.0)	0.282
	Have someone to hold me accountable	57 (27.5)	65 (24.4)	0.445
	Something else	0 (0)	3 (1.1)	0.125
Motivation: automatic and reflective – I would have to. . .	Feel that I want to do it enough (e.g. enjoy eating healthy or exercising)	152 (73.4)	168 (63.2)	0.018
	Feel that I need to do it enough (e.g. believe that my own health is important and feel the need to prioritize self-care)	136 (65.7)	157 (59.0)	0.138
	Believe that it would be a good thing to do (e.g. it will help me cope emotionally or make me feel better)	132 (65.7)	153 (57.5)	0.168
	Believe that it is good for my children (e.g. I am being a good example for my child)	126 (60.9)	180 (67.7)	0.125
	Develop better plans for doing it (e.g. have clearer and better developed plan for eating)	117 (56.5)	129 (48.5)	0.083
	Develop a habit of doing it (e.g. get into a pattern of eating healthy without having to think)	139 (67.1)	173 (65.0)	0.631
	Believe in my ability to do it (e.g. have confidence in my ability to prepare healthy meals)	115 (55.6)	126 (47.4)	0.077
	It would have to fit my cultural and religious beliefs (e.g. beliefs about the type of food to eat when breastfeeding)	41 (19.8)	55 (20.7)	0.815
	Something else	1 (0.5)	0 (0)	0.256

CMPC: cardiometabolic pregnancy complication.

Data are presented as n (%) and were analysed using a Pearson’s chi-square test.

#### Opportunity

There was a significance difference between those women with and without prior cardiometabolic pregnancy complications in choice of the following opportunity factors; have necessary materials (e.g. exercise equipment; 47.3% vs 37.6%, p = 0.033) and have more triggers to prompt me (e.g. have more reminders to exercise at a specific time; 44.0% vs 31.6%, p = 0.006) (Table 3). The three main things women with prior cardiometabolic pregnancy complications suggested they would need to participate in a postpartum lifestyle intervention were to have more time to do it (e.g. create a specific time during the day to exercise; 72.9%), have practical support from others (e.g. help with childcare and chores from partner, family and friends; 58.0%) and have enough money to do it (e.g. earn enough to pay for gym membership; 57.0%). In comparison, women without prior cardiometabolic pregnancy complications suggested they would also have to have more time to do it (72.2%), have enough money to do it (69.8%) and have practical support from others (53.0%). There was a significant difference between women who experienced GDM +/− another cardiometabolic pregnancy complication, women who experienced a cardiometabolic pregnancy complication without GDM and women who did not experience a cardiometabolic pregnancy complication regarding the following opportunity-related behaviour change need; have more triggers to prompt me (40.0% vs 48.0% vs 31.6%, p = 0.011). A significant post hoc comparison was observed between women who had experienced a cardiometabolic pregnancy complication without GDM and women who had not experienced a cardiometabolic pregnancy complication for this opportunity factor (p = 0.011).

#### Motivation

There was a significance difference between those women with and without prior cardiometabolic pregnancy complications for the following motivation factor: feel I want to do it enough (e.g. enjoy eating healthy or exercising; 73.4%, 63.2%, p = 0.018) (Table 3). With respect to motivation, the three upmost things women with prior cardiometabolic pregnancy complications suggested they would need were to feel that they want to do it enough (e.g. enjoy eating healthy or exercising; 73.4%), develop a habit of doing it (e.g. get into a pattern of eating healthy without having to think; 67.1%) and feel that they need to do it enough (e.g. believe that their own health is important and feel the need to prioritize self-care; 65.7%). In comparison, women without prior cardiometabolic pregnancy complications suggested they would have to believe that it is good for their children (e.g. I am being a good example for my child; 67.7%), develop a habit of doing it (65.0%) and feel that they want to do it enough (63.2%). There were no significant differences between women who had experienced GDM +/− another cardiometabolic pregnancy complication, women who had experienced a cardiometabolic pregnancy complication without GDM and women who had experienced a cardiometabolic pregnancy complication regarding motivation-related behaviour change needs.

## Discussion

This is the first study to use a framework-based approach to explore and compare preferred intervention characteristics (based on the TIDieR checklist) and behaviour change needs (based on the COM-B system) of women with or without prior cardiometabolic pregnancy complications for engagement in postpartum lifestyle interventions.

We report subtle differences in preferred intervention content between groups. A significantly higher portion of women with prior cardiometabolic pregnancy complications desired intervention content to enhance knowledge including on women’s health, diet, preventing T2D and CVD, exercise and monitoring progress. The preference for content on preventing cardiometabolic disease may be related to these women being aware of their increased future T2D and CVD risk. Alternatively, they may have dissatisfaction with this information received from healthcare professionals. This is consistent with some women with prior GDM and PE reporting feeling abandoned by the healthcare system and unsupported in managing cardiometabolic disease risk, general health and wellbeing postpartum.^29–34^ Where lifestyle support is provided, some women report receiving competing information from multiple healthcare professionals and a lack of empathetic and patient-centred and culturally sensitive information.^32,35,36^ Postpartum lifestyle interventions for these women should therefore include appropriate information, skill development and risk communication specific to T2D and CVD risk awareness and risk reduction.

Both groups preferred intervention content to be on women’s health and mental health with monitoring of progress and social support for their health. A lack of social support from partners, family, friends and healthcare professionals is a commonly reported barrier to engagement in healthy lifestyles postpartum, with its presence a commonly reported facilitator^12^ associated with improved physical activity, diet and depressive symptoms.^37^ In addition, some women with GDM desire access to peer support groups to aid in postpartum lifestyle management.^38^ Regarding mental health, previous studies report a higher portion of women who experienced PE had higher levels of depression 6 months postpartum and were more likely to describe their birth as a traumatic event compared to women who experienced a normotensive pregnancy.^39^ GDM was similarly associated with increased postpartum anxiety and depression.^40^ While lifestyle interventions typically focus on diet and physical activity, good mental health is an integral part of a healthy lifestyle. Poor mental health is also a barrier to engagement in a healthy diet and physical activity in women with prior GDM.^15^ This emphasizes the need to consider a holistic approach to lifestyle interventions incorporating mental health components.

We report the majority of women were interested in postpartum lifestyle programmes and would prefer them to be delivered by someone with expertise in women’s health, such as healthcare professionals. This is consistent with women having regular interactions with healthcare professionals during pregnancy,^41^ which likely facilitates building of trust and rapport. Furthermore, postpartum lifestyle interventions delivered by healthcare professionals are more effective for weight loss.^25^ Over two-thirds of all women also preferred the setting to be either their maternal and child health nurse visits or online and for content to be delivered online or via one-on-one face-to-face consultations. This coincides with an increased popularity and use of digital and eHealth technologies,^42^ which are both highly accepted among postpartum women^43^ and effective in facilitating postpartum weight management.^42^ In addition, eHealth interventions can provide more flexibility and address some barriers to engagement for postpartum women including time commitments.^43,44^ With > 85% of postpartum women owning a smartphone with Internet access,^45^ engaging high-risk women in postpartum healthy lifestyle interventions will likely benefit from face-to-face consultations aided by technology-based engagement, information and resources.^46,47^

We report both groups of women would prefer postpartum lifestyle interventions to be initiated between 7 weeks to 3 months postpartum and last ~1 year. There is a lack of consensus regarding optimal initiation and duration of postpartum lifestyle interventions.^41^ A recent systematic review of women with previous GDM suggests those initiated within 6 months of birth are more effective in reducing future T2D risk than those commenced later,^48^ coinciding with the current participants preferences. However, attrition in lifestyle interventions is higher in the early postpartum period (6-week compared to 6-month postpartum follow-up visit),^17^ indicating challenges for early commencement. The majority of women preferred the intervention to be monthly sessions of 15–30 min, consistent with lack of time as a common barrier to engagement in postpartum lifestyle interventions,^12^ and the most prevalent opportunity-related behaviour change factor for engagement in this study. Shorter session and programme lengths and less frequent sessions will likely engage, recruit and retain more women than longer programmes with more frequent and longer sessions. Further research should investigate how to achieve desired intervention preferences without compromising intervention effectiveness. Of interest, we note significantly more women with prior cardiometabolic complications preferred lower frequency (3 monthly) appointments. This could indicate the need for a period of adjustment before initiating lifestyle management for some of these high-risk women, potentially related to factors including greater physical and mental health demands of complicated pregnancies.^39,40,49^

With respect to behaviour change needs, there were no significant differences between the two groups regarding capability-related factors. However, regarding opportunity, significantly more women with prior cardiometabolic pregnancy complications felt they needed to have the necessary materials (e.g. exercise equipment) and more triggers to prompt them (e.g. exercise reminders), and regarding motivation, significantly more women with prior cardiometabolic pregnancy complications felt they had to want to do it enough (e.g. enjoy eating healthy or exercising), to engage in postpartum lifestyle interventions. It is possible these women already have insight into the need to change their behaviours and require more resources and support and something more tailored to their preferences to engage. Previous research using the COM-B in multi-ethnic postpartum GDM women’s engagement in healthful behaviours similarly reported beliefs about consequences and the necessity of health behaviours as a key motivation factor influencing engagement.^15^ Additional research identifying barriers to reducing diabetes risk through lifestyle change following GDM reports women acknowledging that being held accountable and having more resources (e.g. free exercise facilities, healthy recipes, home exercise equipment or videos) would help them to be healthier.^36^ The lack of differences between the two groups regarding other behaviour change needs suggests barriers to lifestyle engagement faced are similar. Emphasizing facilitators and practically addressing these barriers is crucial in developing a lifestyle intervention in which participants can implement healthful behaviours in an engaged, sustainable, self-sufficient and long-term manner.

The strengths of this study include being the first to compare intervention characteristic preferences and behaviour change needs of women with and without a range of prior cardiometabolic pregnancy complications. This extends prior research with women in the general population or those with GDM to explore other cardiometabolic pregnancy complications. Due to the smaller sample size in these groups, it was not possible to perform subgroup analysis for each specific pregnancy complication which is warranted in future research. We report a high prevalence of women born outside of Australia or from a cultural or ethnic background separate to Caucasian. This represents the multicultural society of present-day Australia,^50^ increasing the findings transferability and generalizability to the Australian population. However, since the sample is mostly well educated, working women with a middle-to-high income, it does not focus on women where health equity is likely compromised, for example those with low income, low education levels and recent migrants and refugees. Future research should consider further amplifying these women’s voices where possible in addition to better understand how intervention preferences and behaviour change needs may differ depending on cultural background to enhance patient-centred care and the cultural appropriateness of postpartum intervention content and delivery. Such differences have previously been explored in postpartum women in general, with subtle differences observed in preferences of women with Oceanian background compared with Asian background on intervention characteristics including later initiation, less frequent and shorter duration sessions and consideration of the cultural relevance of food and health practices.^51^ We were not able to explore these differences further by cardiometabolic pregnancy complication due to the limited sample size in stratified groups and note this as an important area for future research. Future research should also investigate barriers to accessing lifestyle interventions for women and how to address these. We acknowledge the original survey was only tested for acceptability, not reliability and validity. We also acknowledge that no prior sample size calculations were conducted, due to no prior research in this area.

## Conclusion

Postpartum women with prior cardiometabolic pregnancy complications are interested in engaging in lifestyle interventions postpartum. They have unique preferences regarding intervention characteristics and behaviour change needs which may influence their engagement. These include the intervention being centred around the topic of women’s health and cardiometabolic health, diet and exercise after birth, providing accountability, assisting women in understanding their risks and the importance of engaging in a healthy lifestyle postpartum and supporting and motivating them to engage within their current responsibilities. Consideration of these is crucial for tailored postpartum lifestyle intervention design and implementation success to overcome barriers and enhance facilitators to engagement. This will improve engagement of these high-risk women in postpartum lifestyle interventions, in the hope of improving their overall health and wellbeing and their risks of future cardiometabolic disease.

## Supplemental Material

sj-docx-1-whe-10.1177_17455057241247748 – Supplemental material for Preferred lifestyle intervention characteristics and behaviour change needs of postpartum women following cardiometabolic pregnancy complications Supplemental material, sj-docx-1-whe-10.1177_17455057241247748 for Preferred lifestyle intervention characteristics and behaviour change needs of postpartum women following cardiometabolic pregnancy complications by Elaine K Osei-Safo, Siew Lim, Maureen Makama, Mingling Chen, Helen Skouteris, Frances Taylor, Cheryce L Harrison, Melinda Hutchesson, Christie J Bennett, Helena Teede, Angela Melder and Lisa J Moran in Women’s Health

sj-docx-2-whe-10.1177_17455057241247748 – Supplemental material for Preferred lifestyle intervention characteristics and behaviour change needs of postpartum women following cardiometabolic pregnancy complications Supplemental material, sj-docx-2-whe-10.1177_17455057241247748 for Preferred lifestyle intervention characteristics and behaviour change needs of postpartum women following cardiometabolic pregnancy complications by Elaine K Osei-Safo, Siew Lim, Maureen Makama, Mingling Chen, Helen Skouteris, Frances Taylor, Cheryce L Harrison, Melinda Hutchesson, Christie J Bennett, Helena Teede, Angela Melder and Lisa J Moran in Women’s Health

## Declarations

**Ethics approval and consent to participate:** The online survey study was approved by the Monash University HREC (Project no. 29273) on 21 September 2021. Written informed consent to participate was obtained from all participants involved in the study.

**Consent for publication:** Written informed consent for publication was obtained from all participants involved in the study.

**Availability of data and material:** The original survey is included as a supplementary file. The original data presented in this study are available on request from the corresponding author. The data are not publicly available because they are potentially identifiable.
