# Supplementary figures and images for: Preferred lifestyle intervention characteristics and behaviour change needs of postpartum women following cardiometabolic pregnancy complications
Source: Womens Health (Lond). 2024 Jul 26;20:17455057241247748. doi: 10.1177/17455057241247748 (PMC11282569; doi:10.1177/17455057241247748)

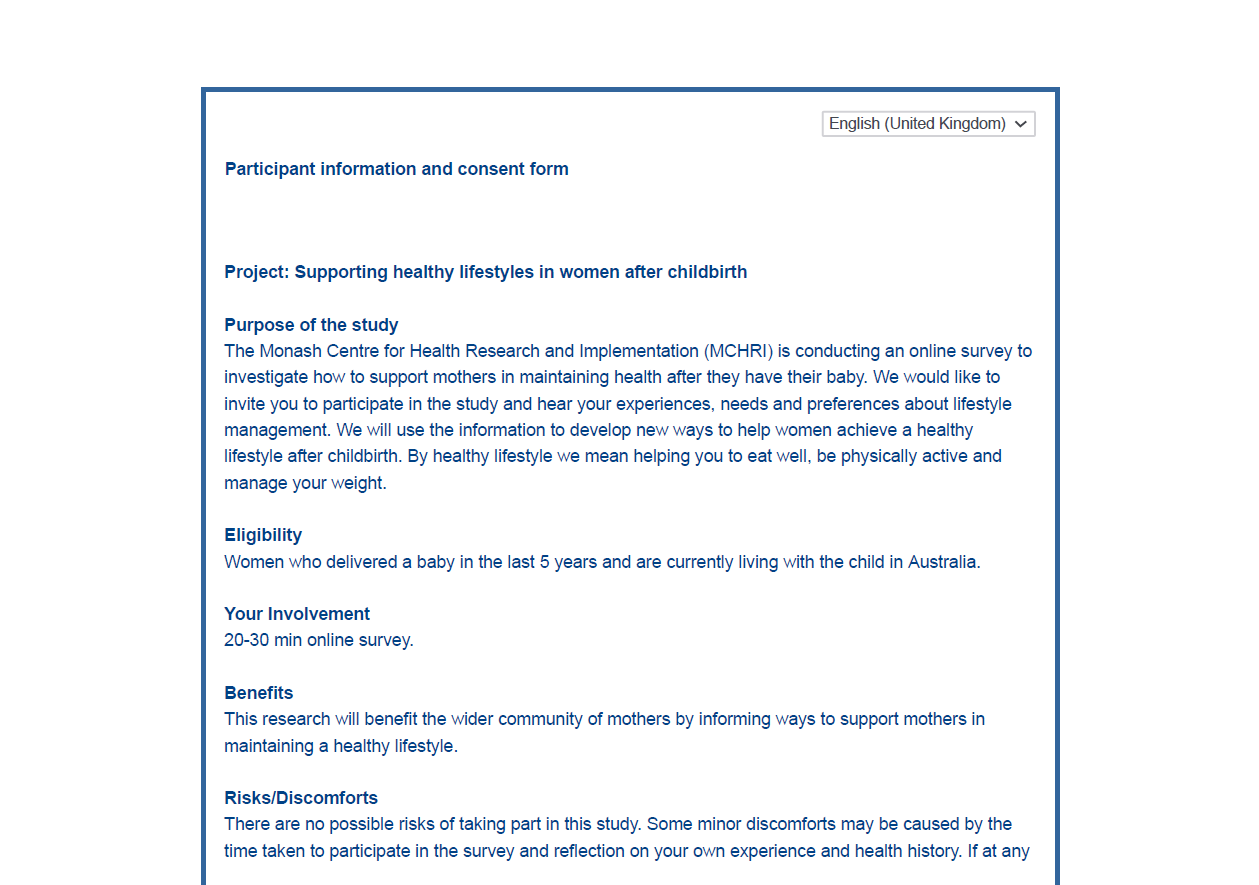


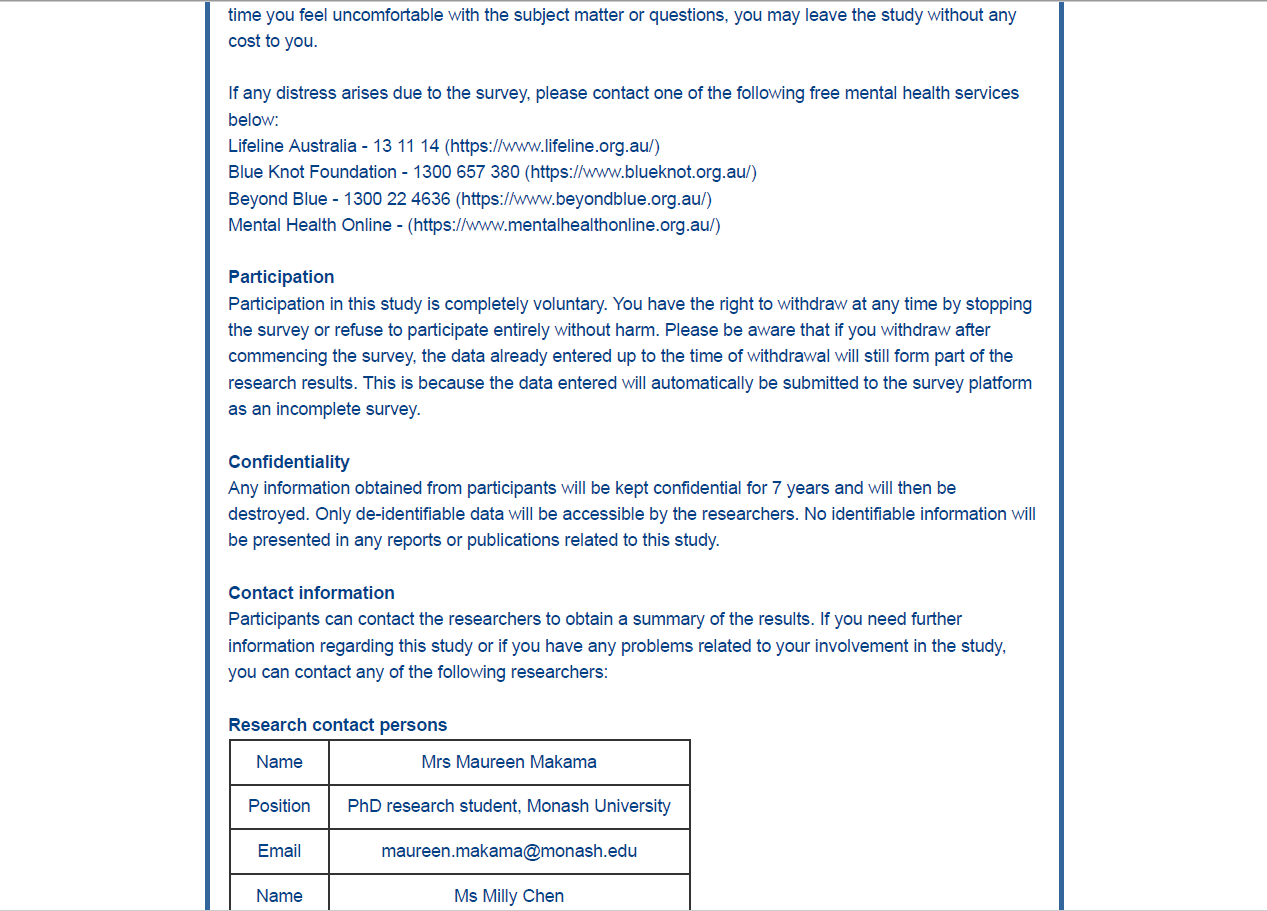


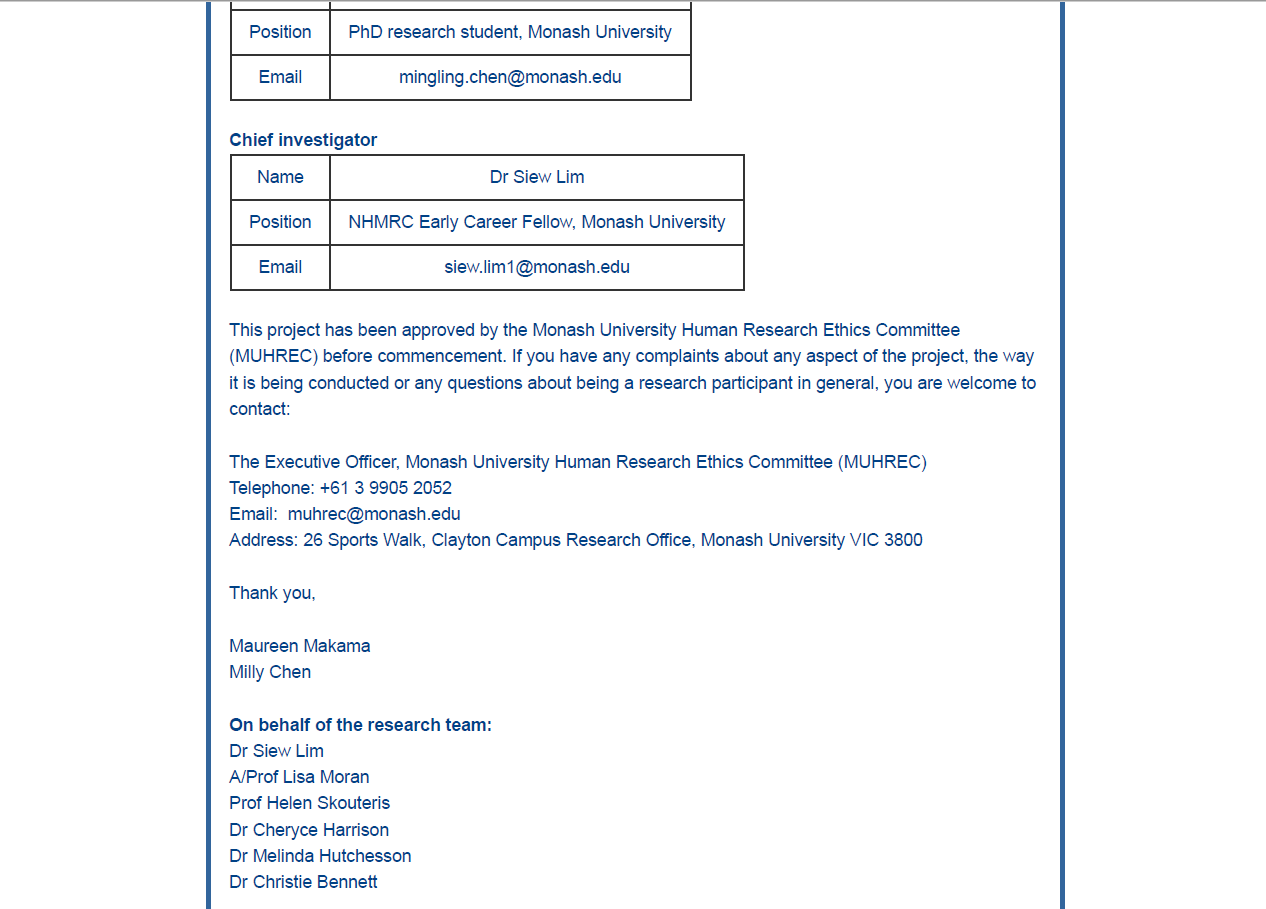


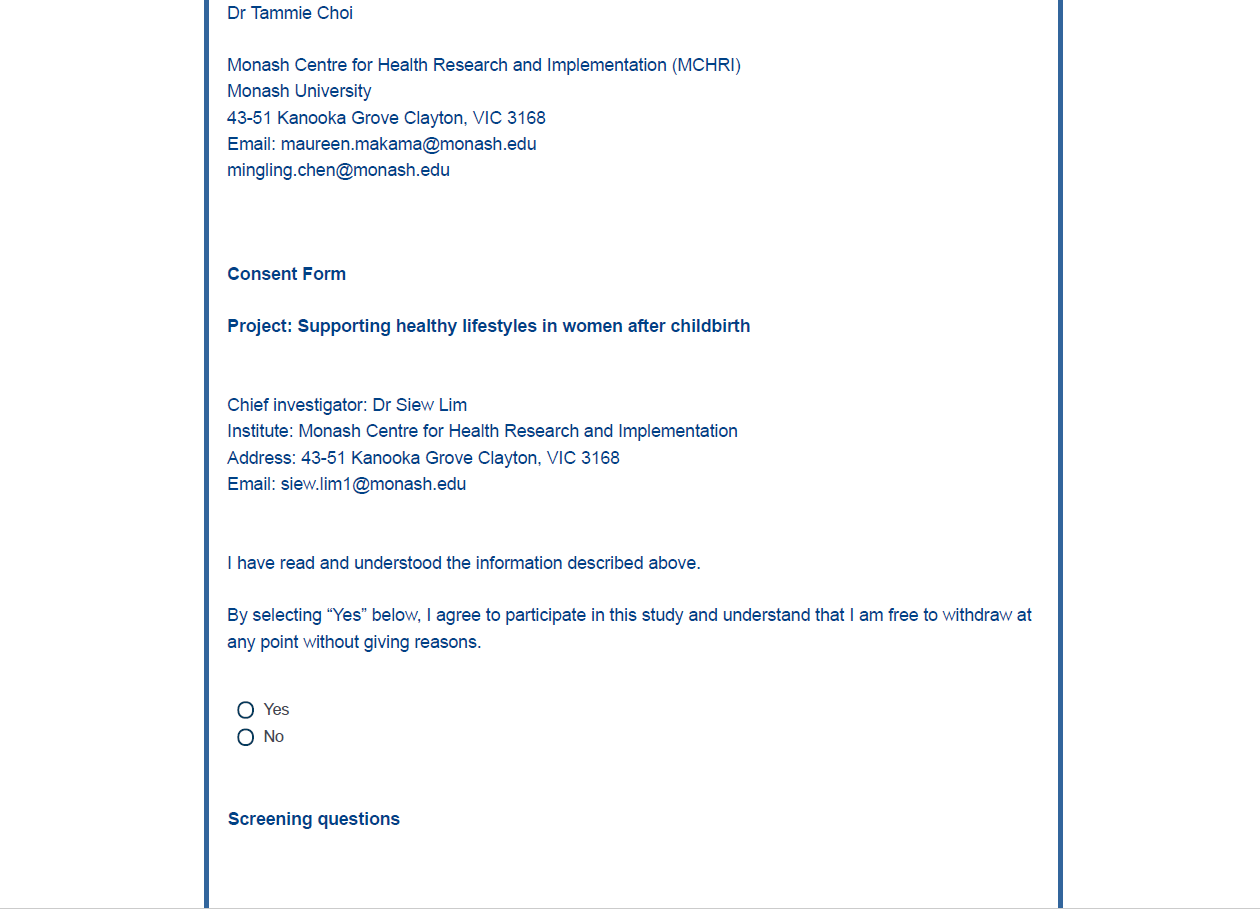


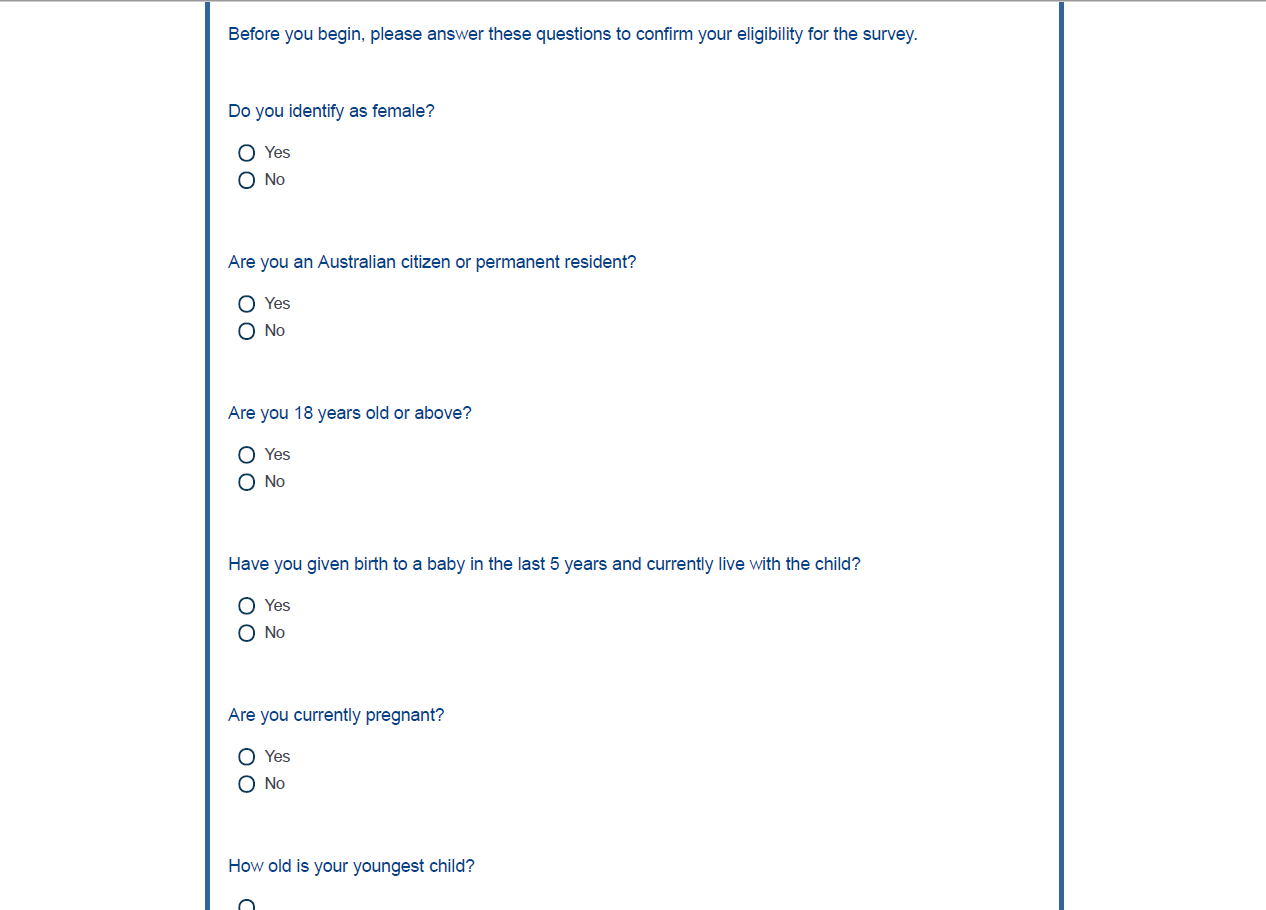


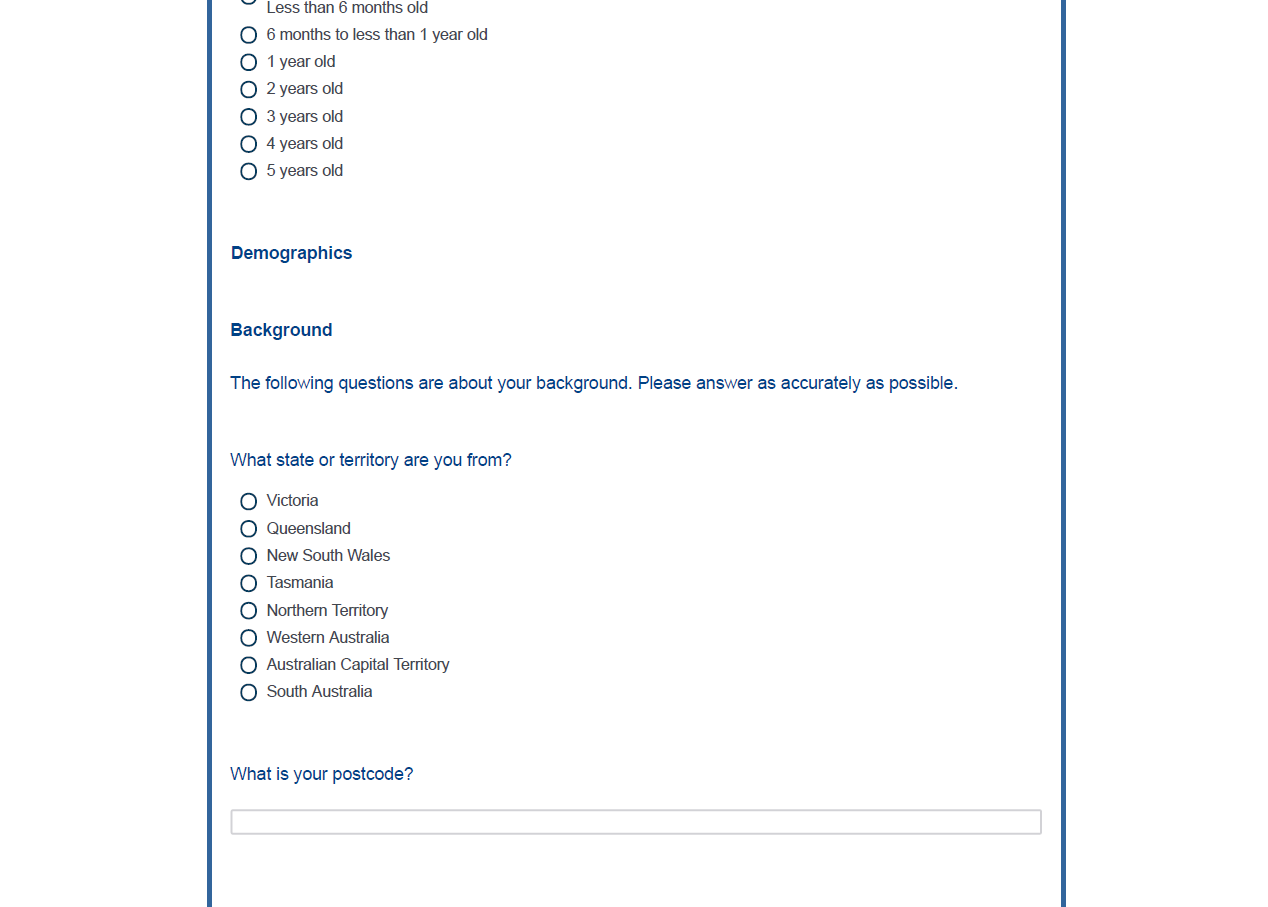


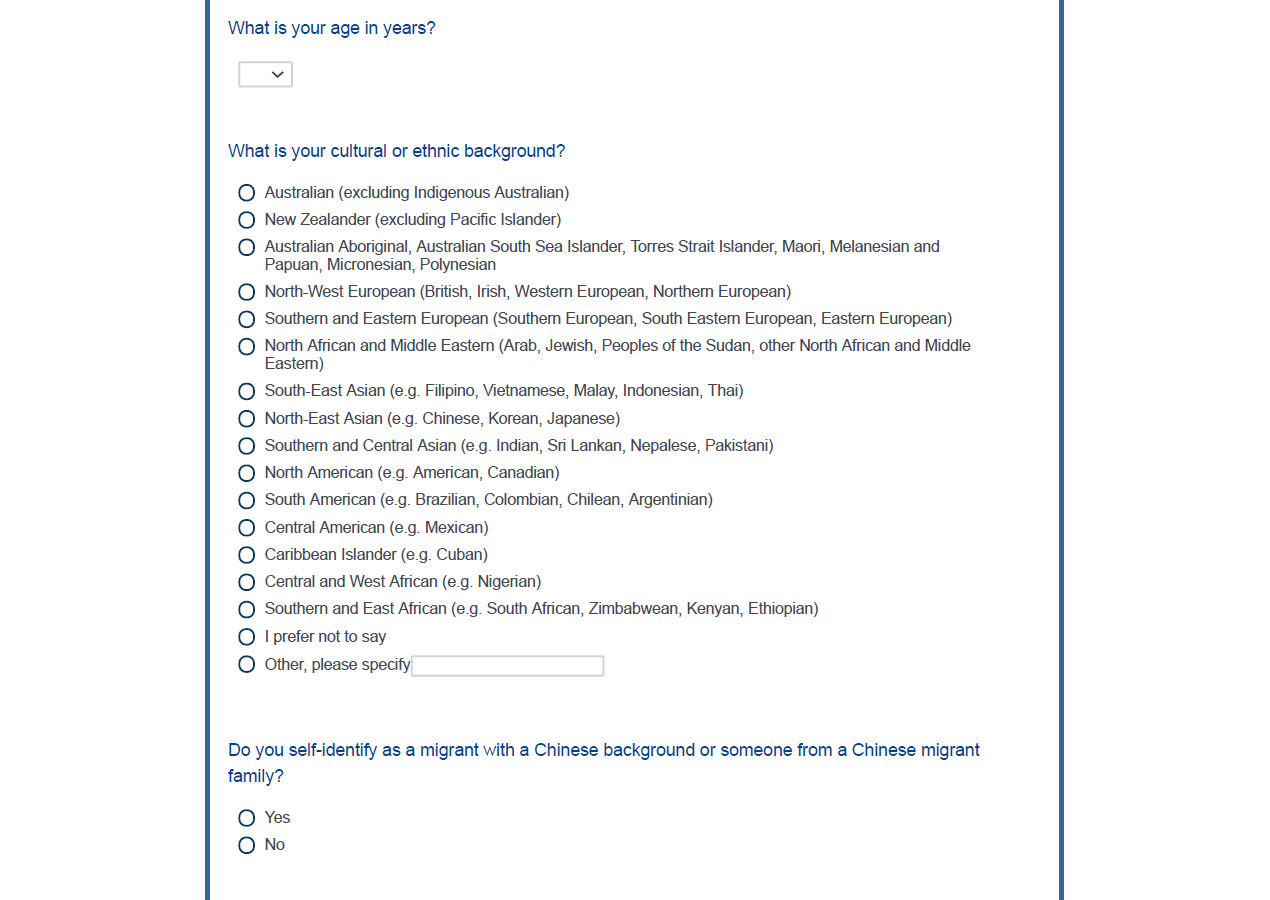


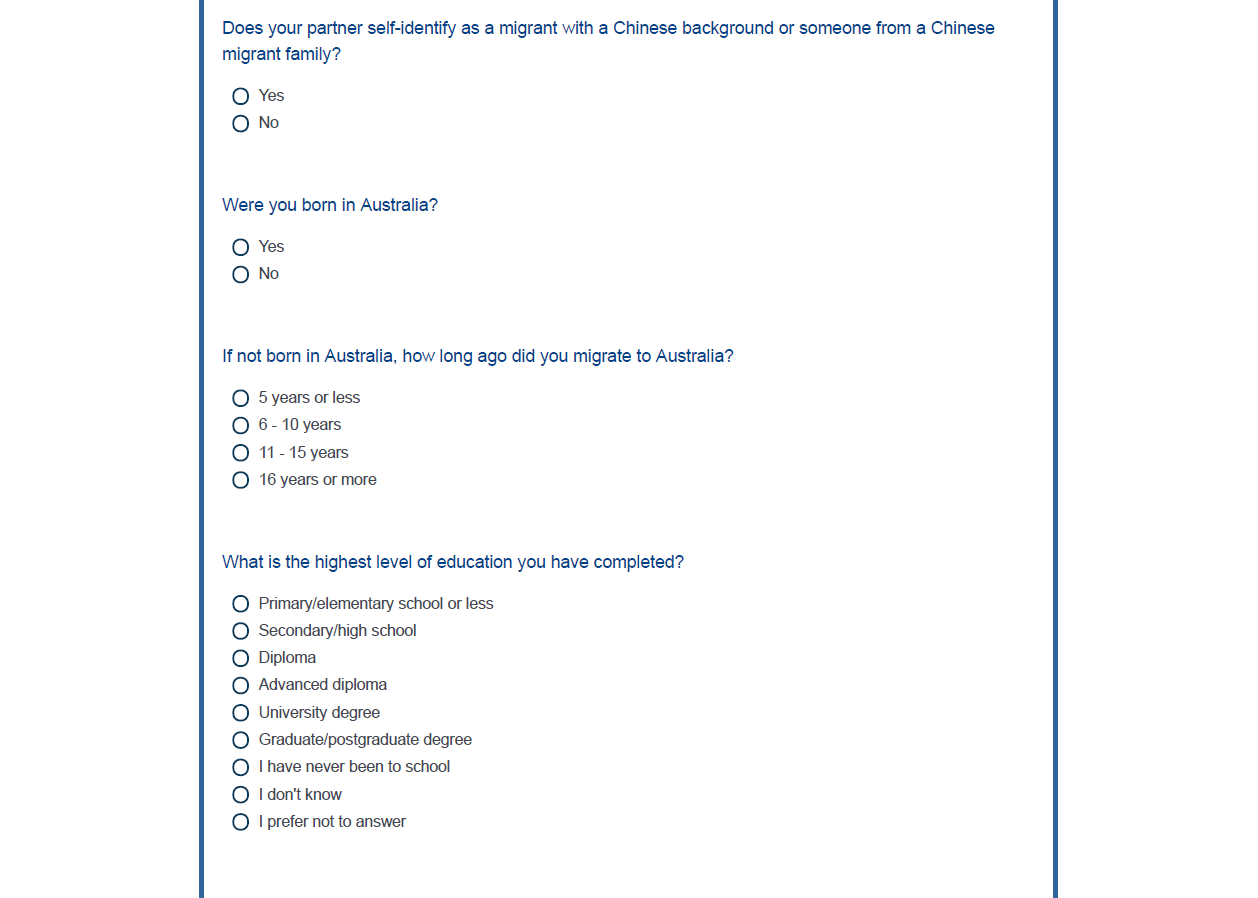


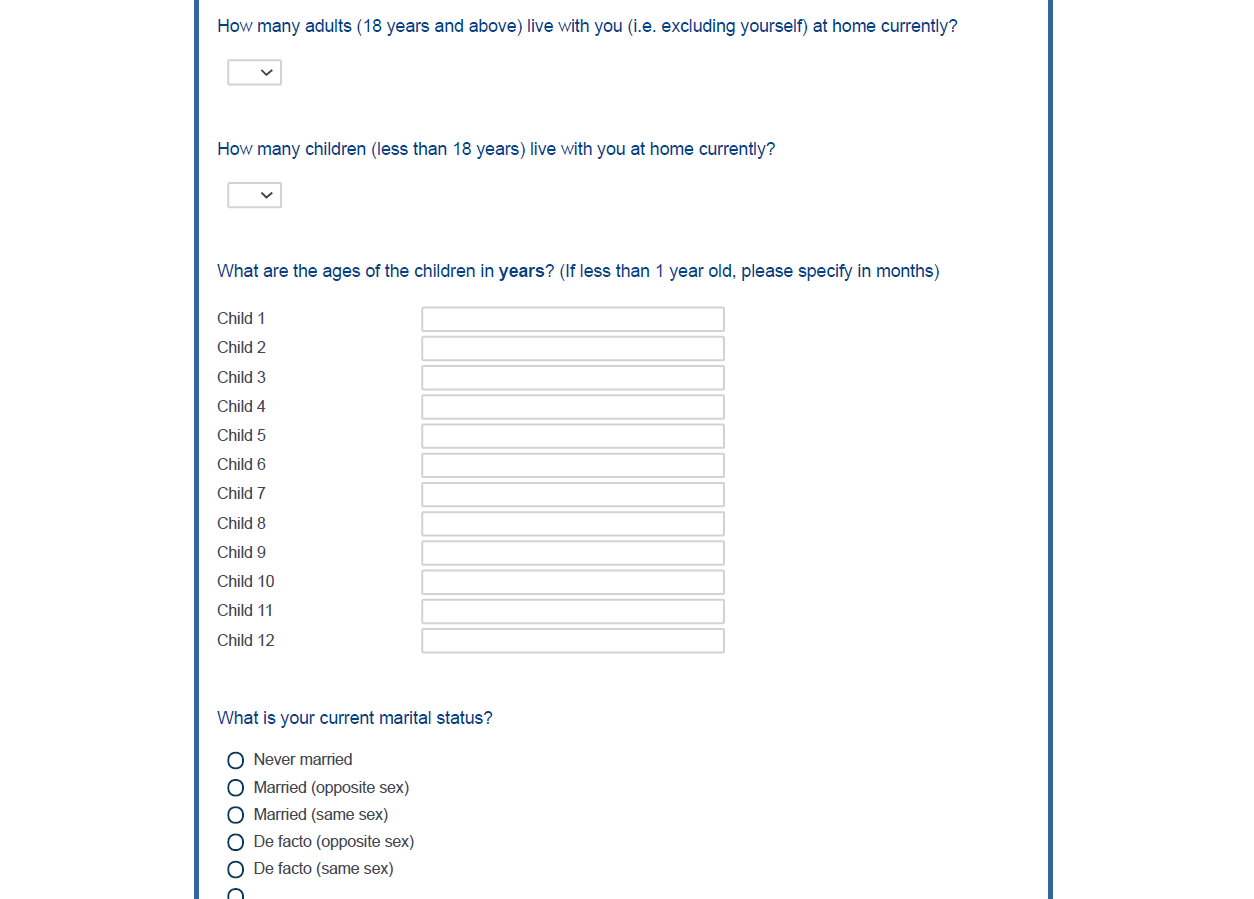


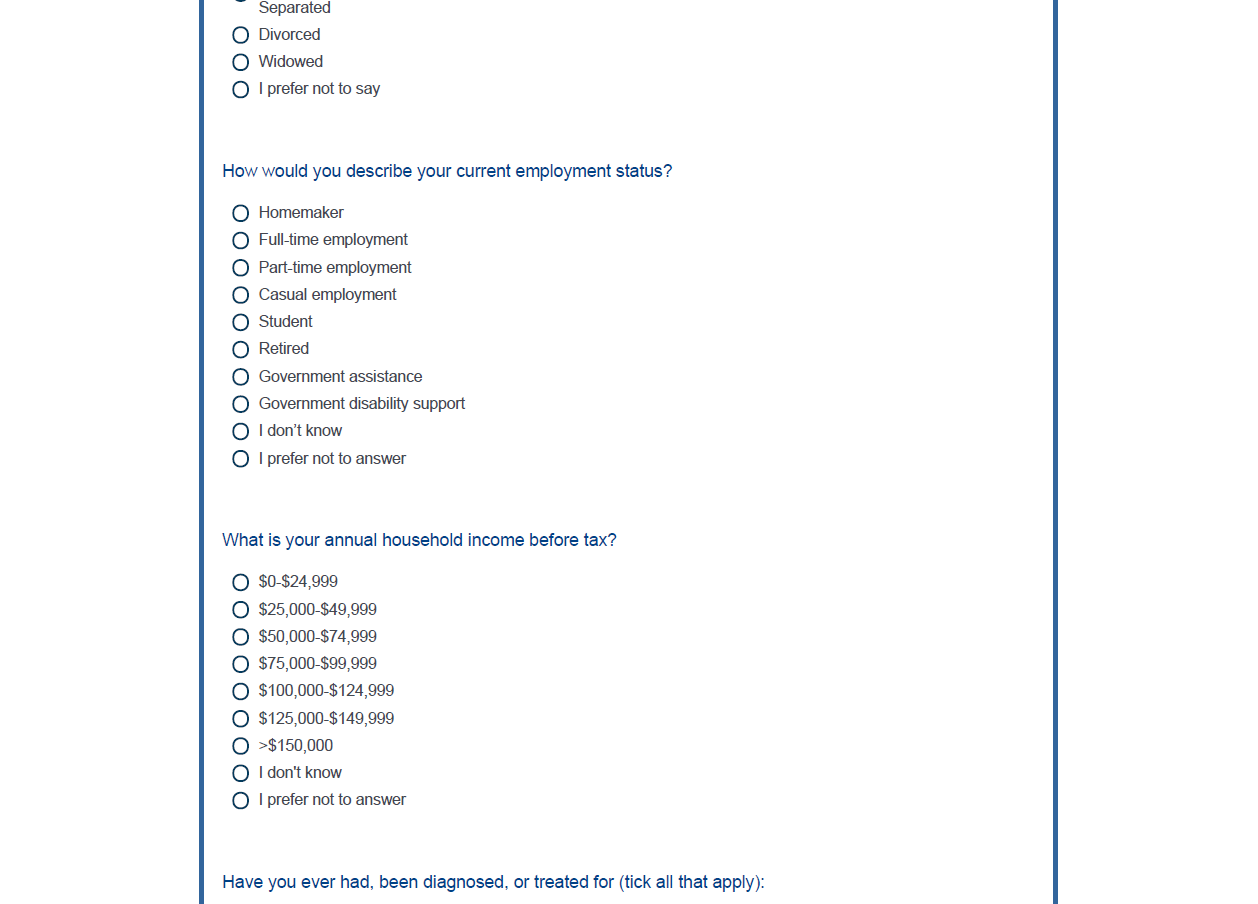


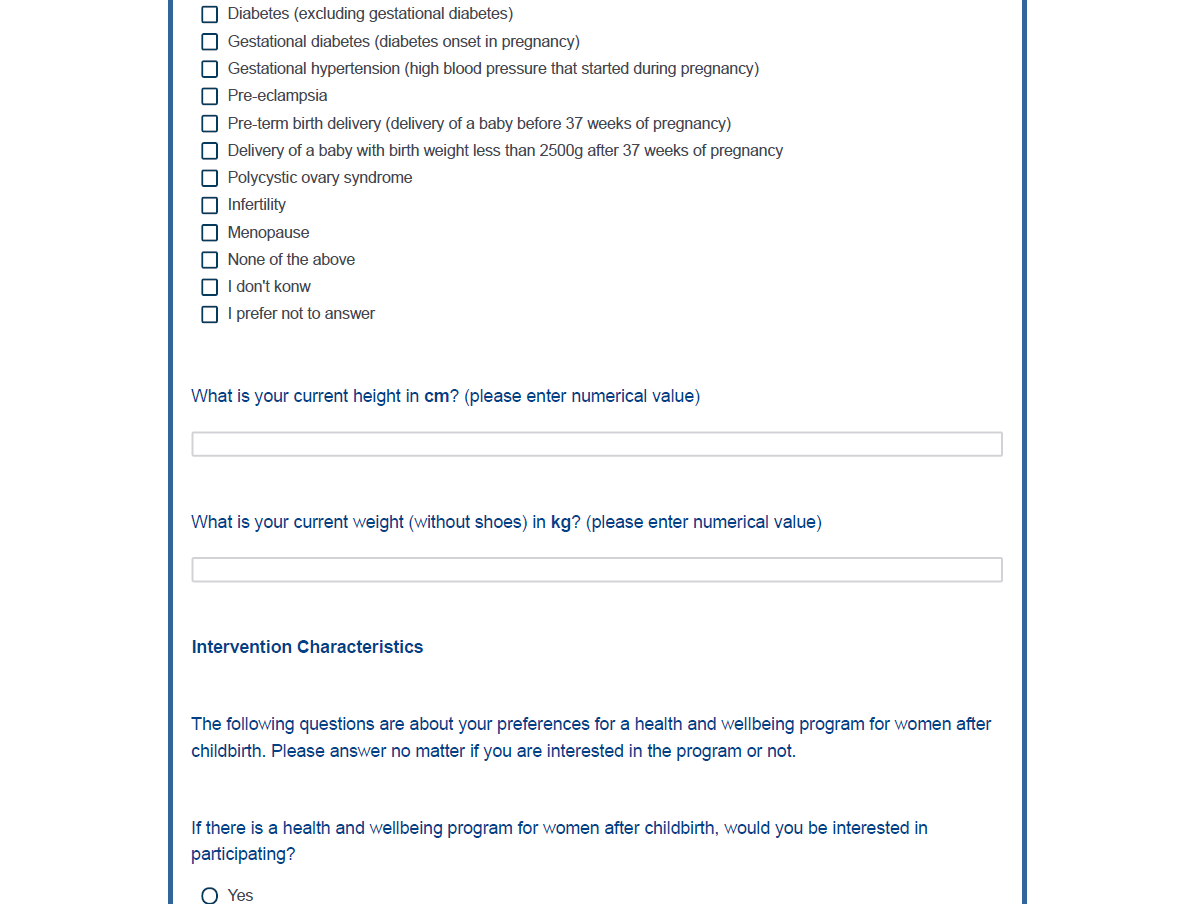


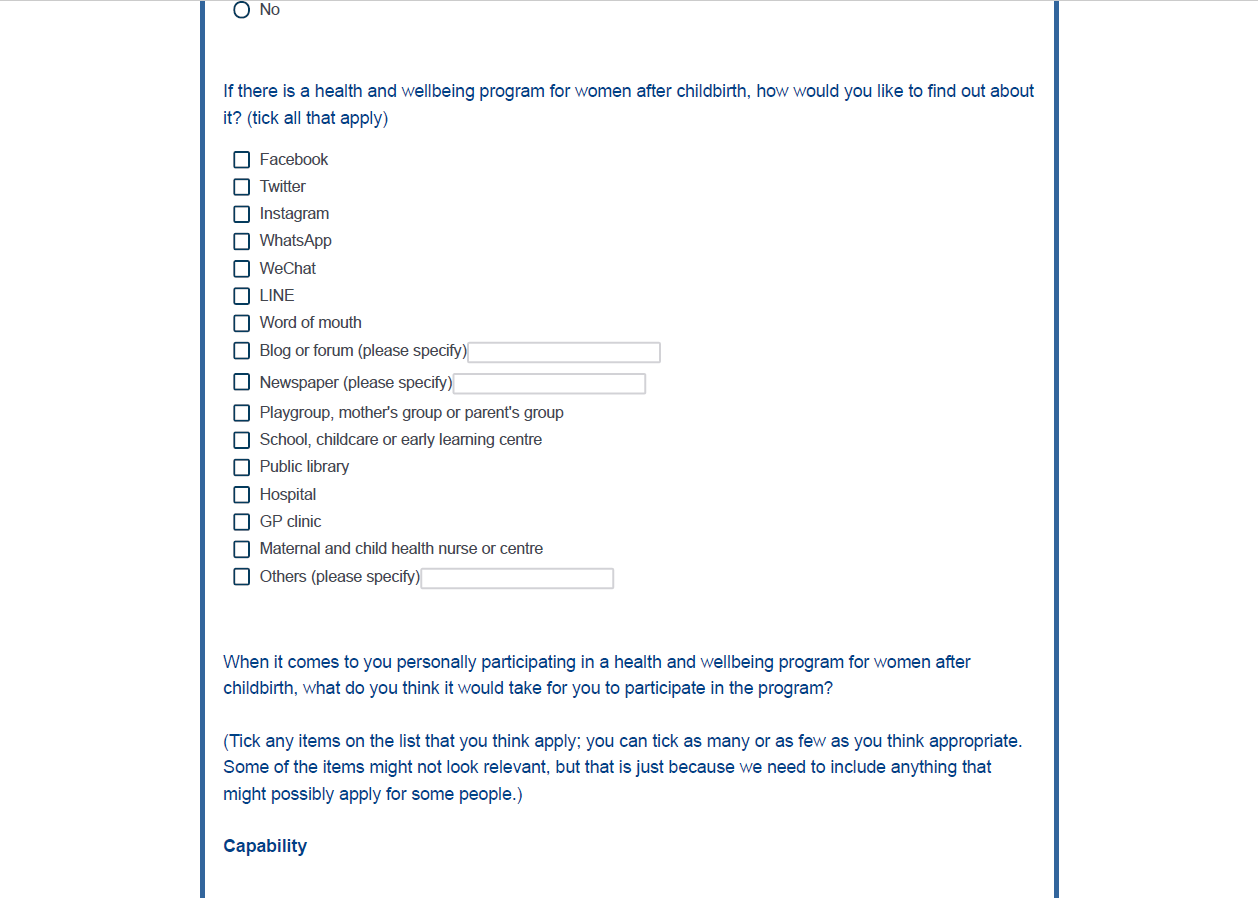


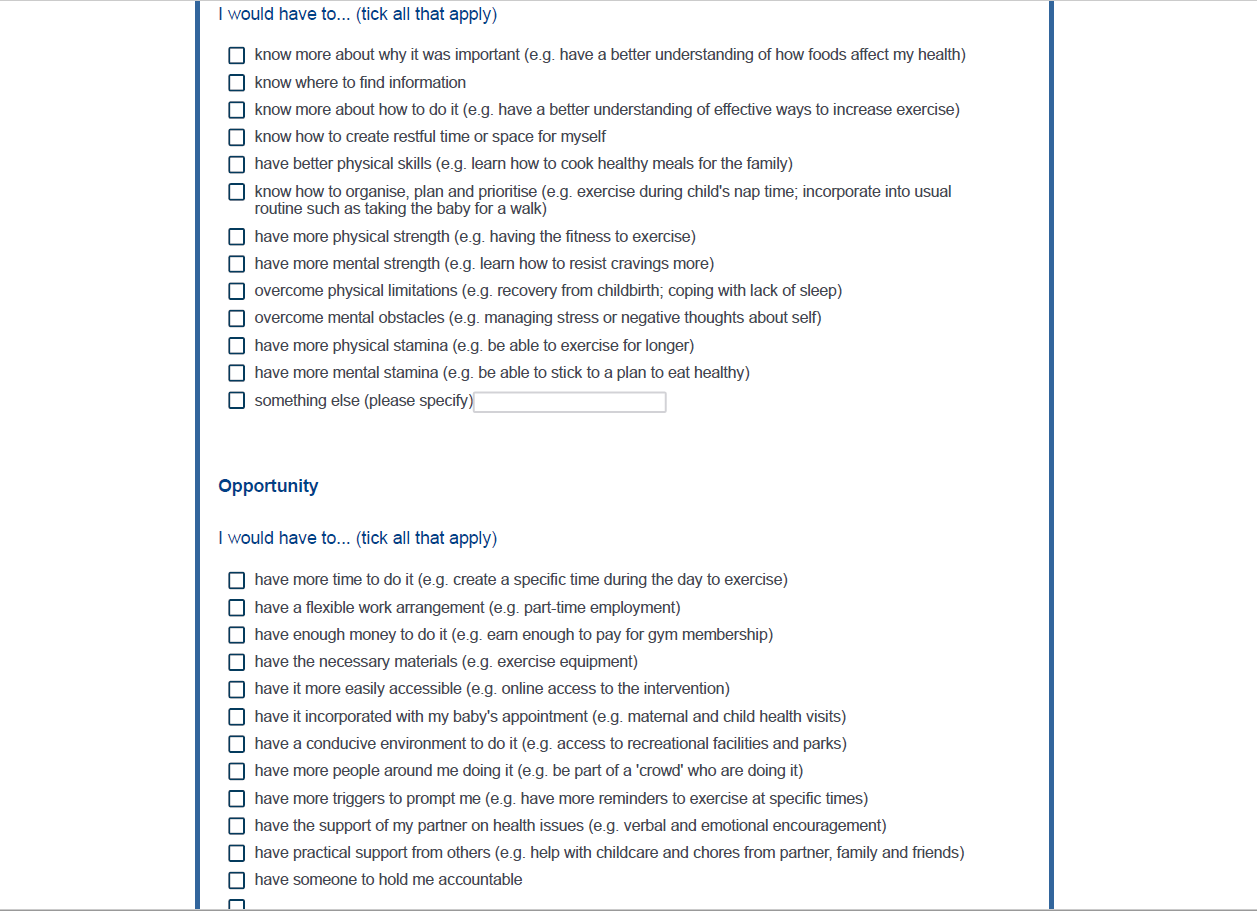


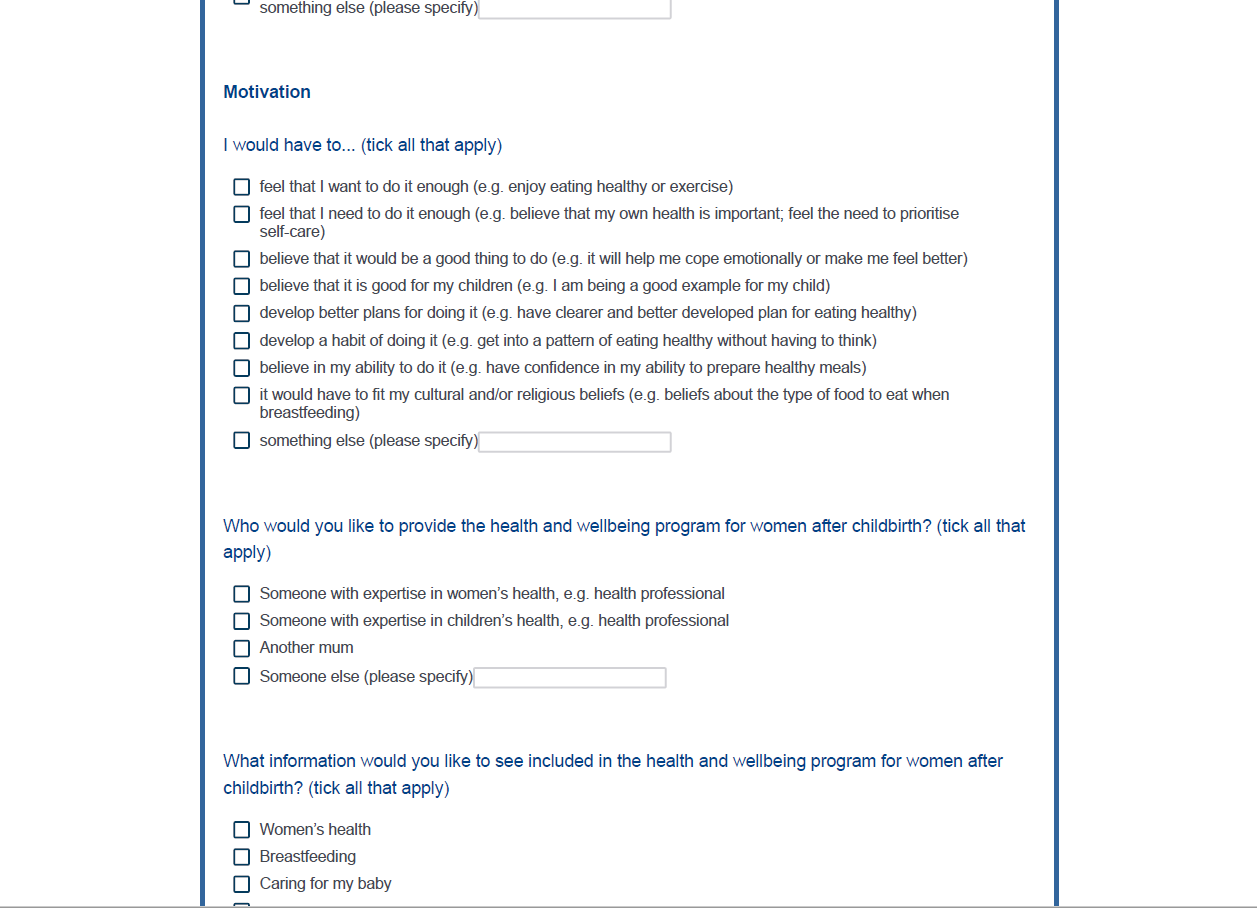


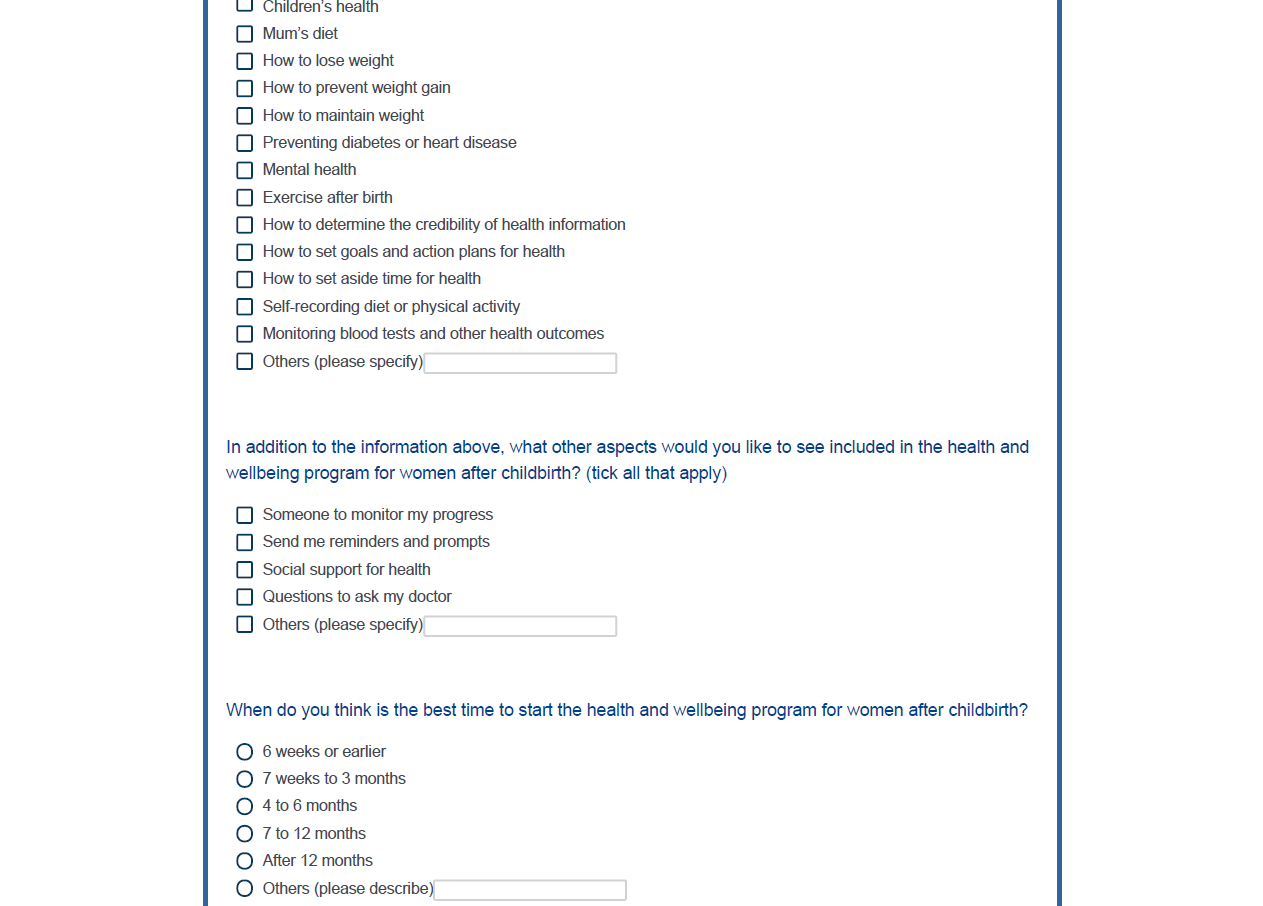


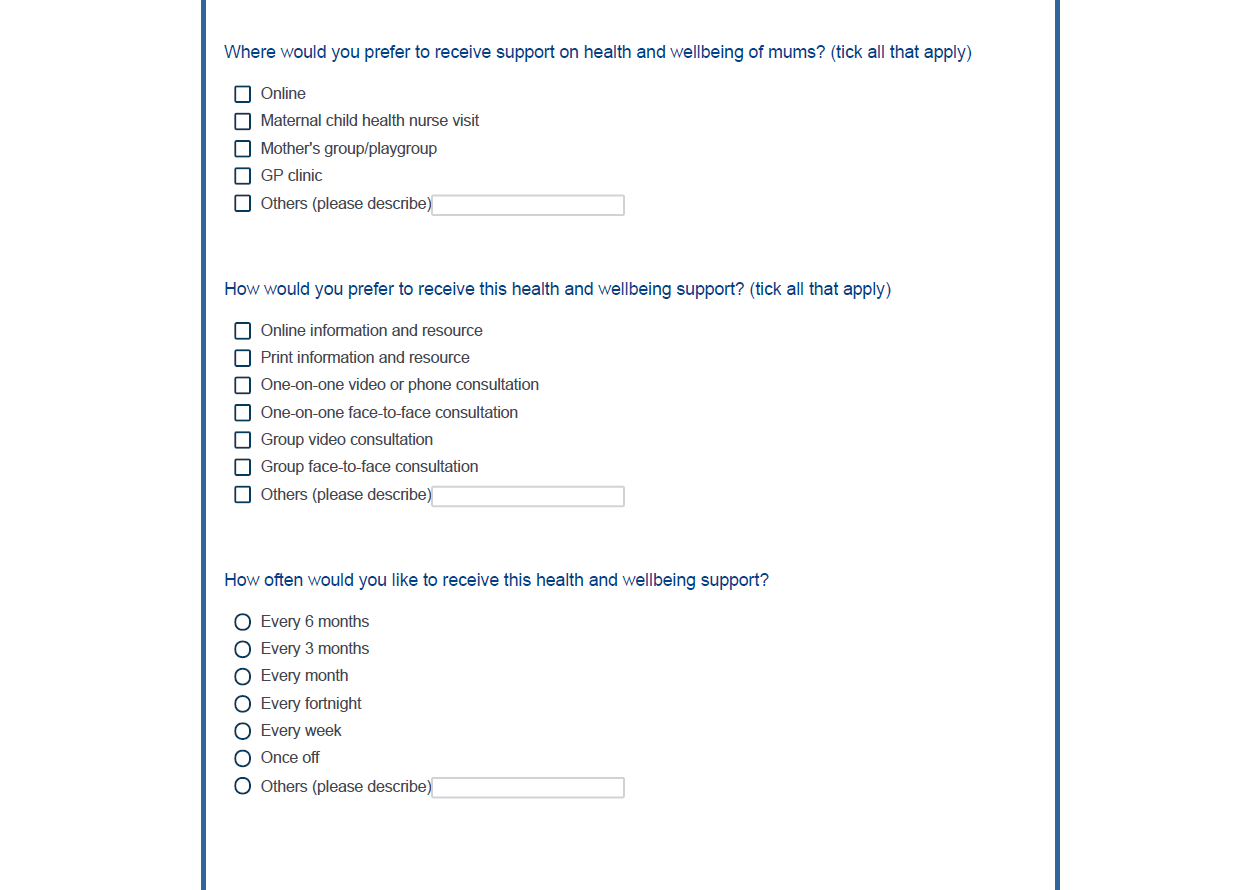


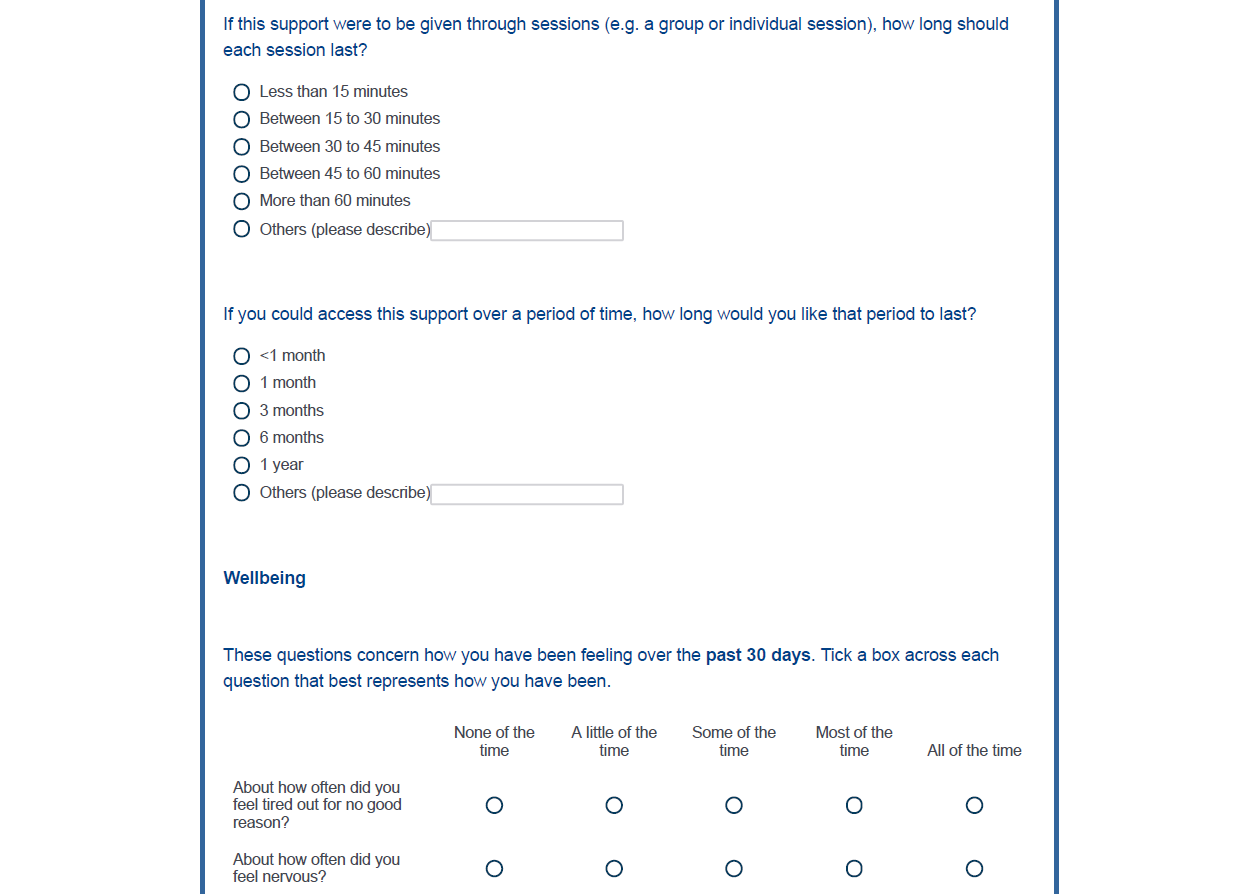


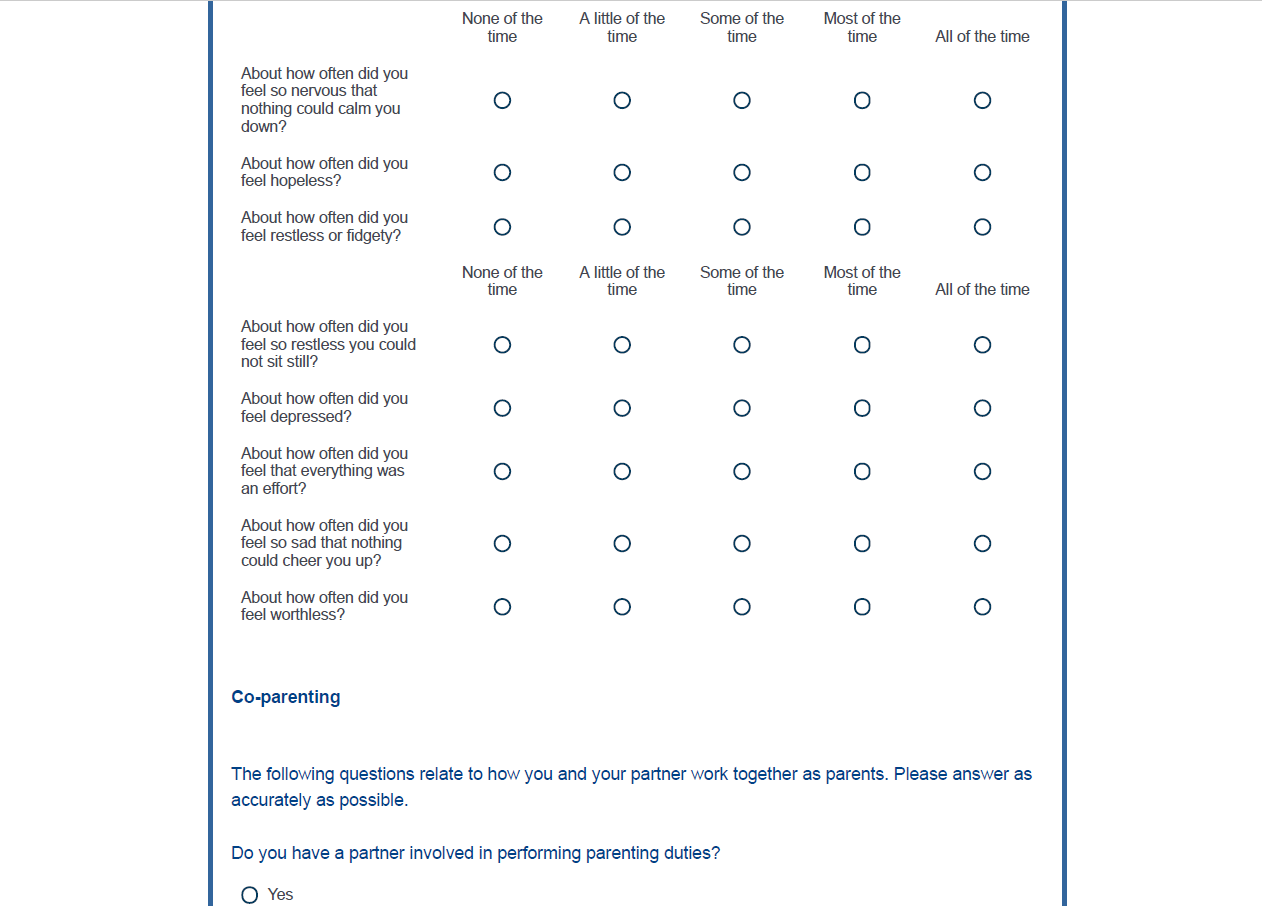


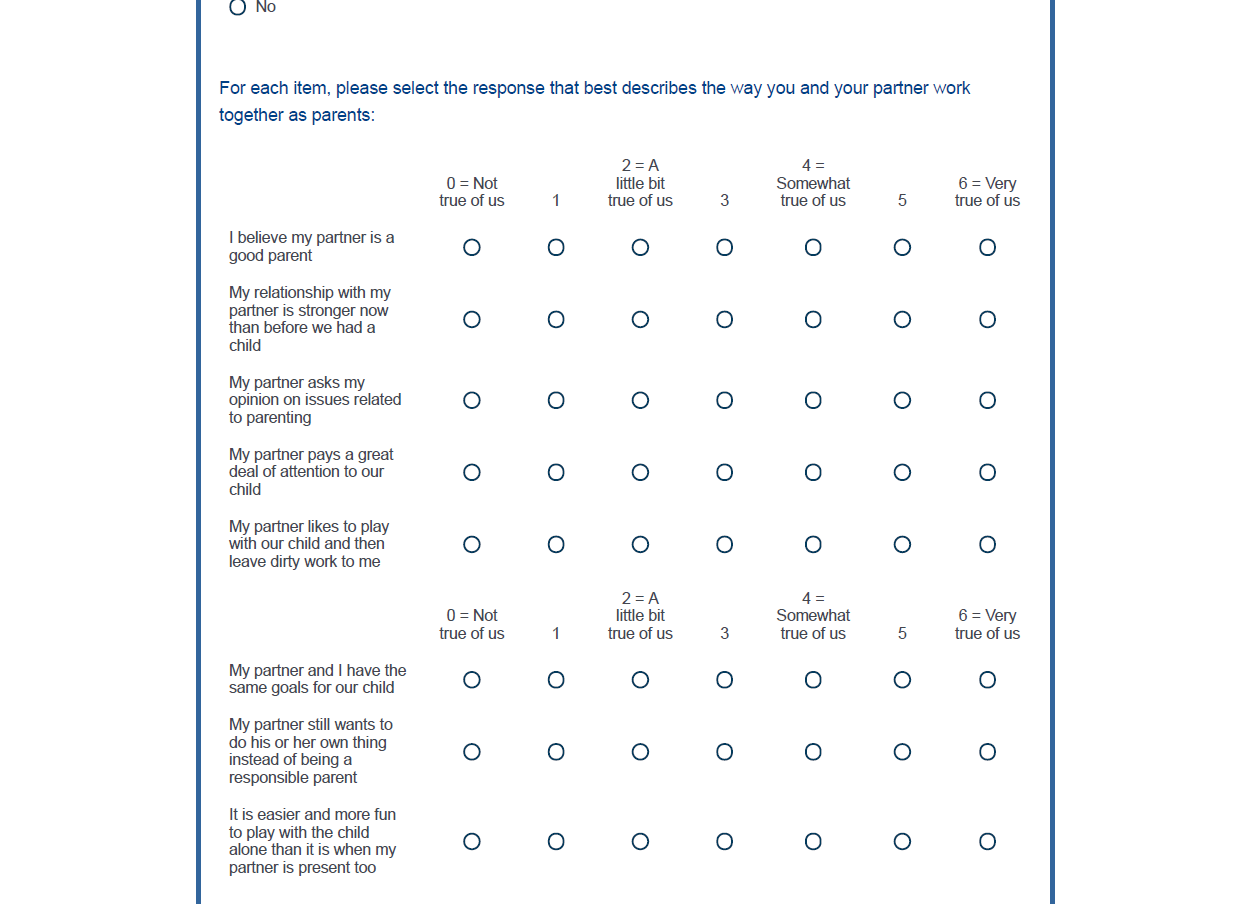


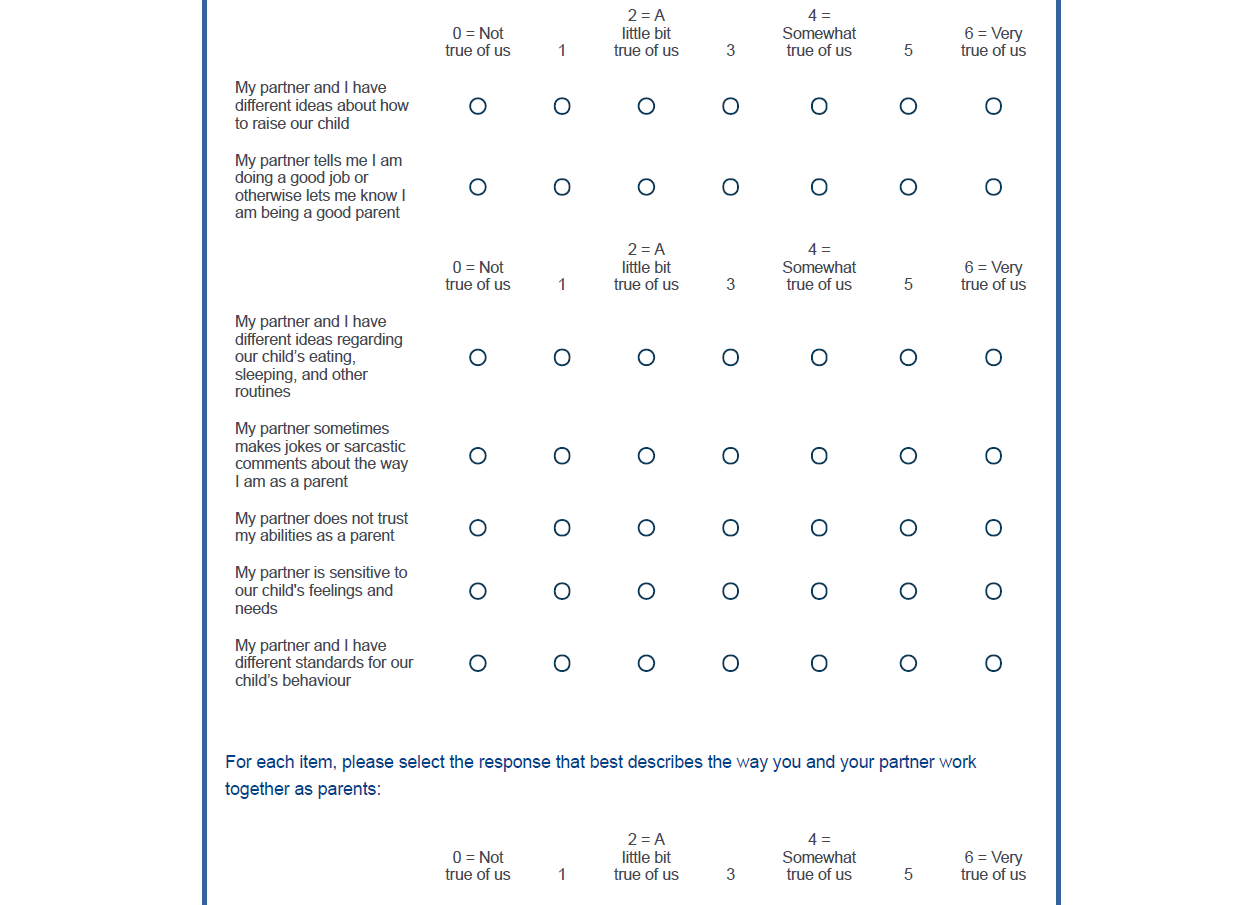


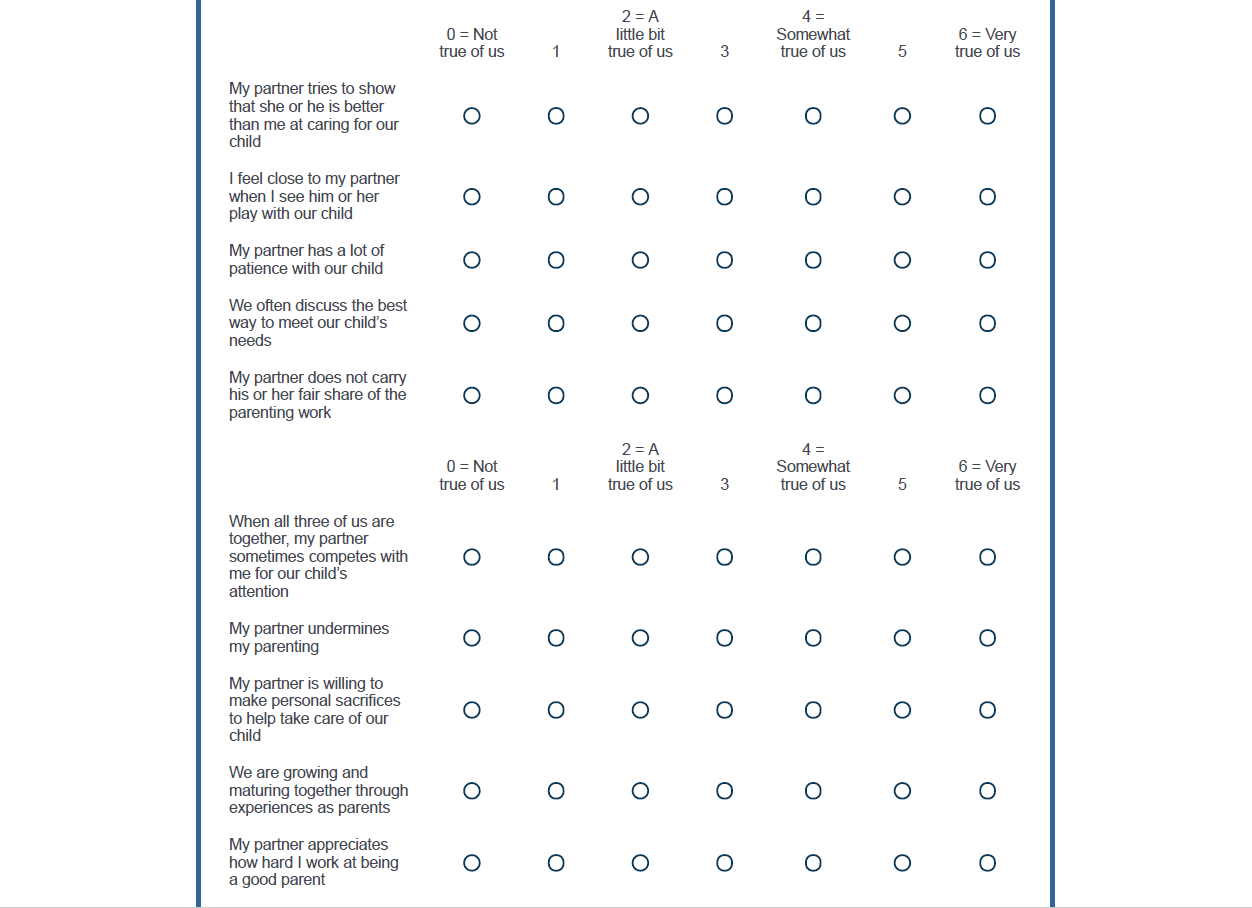


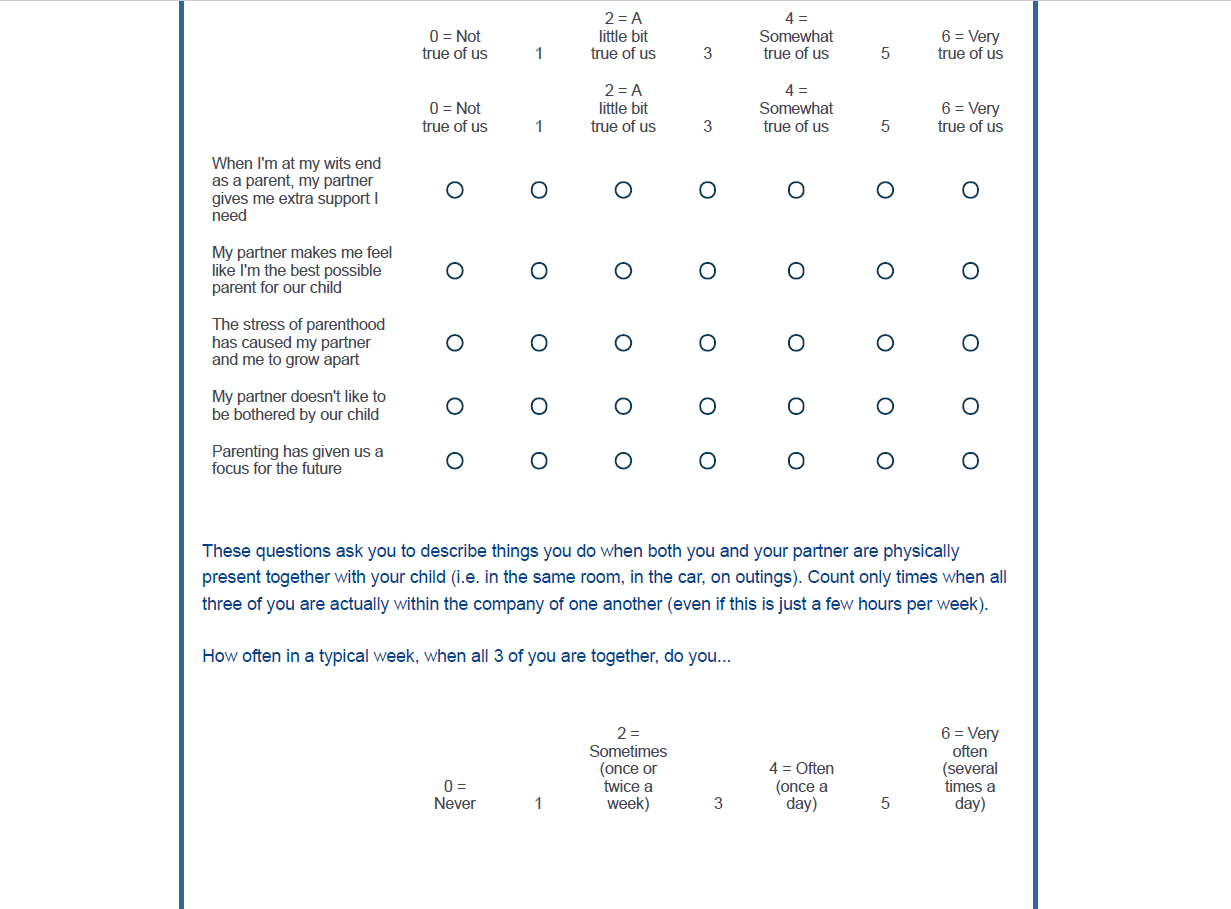


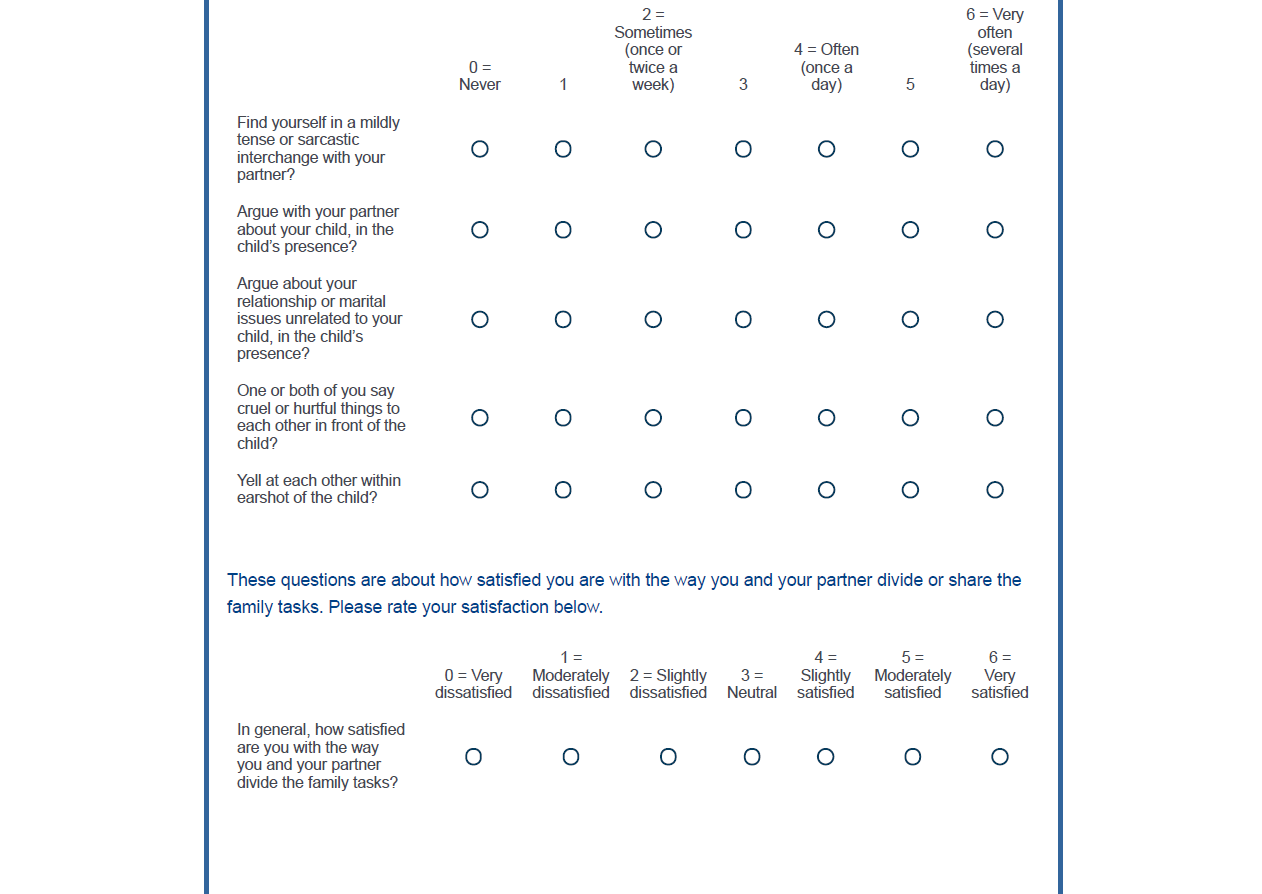


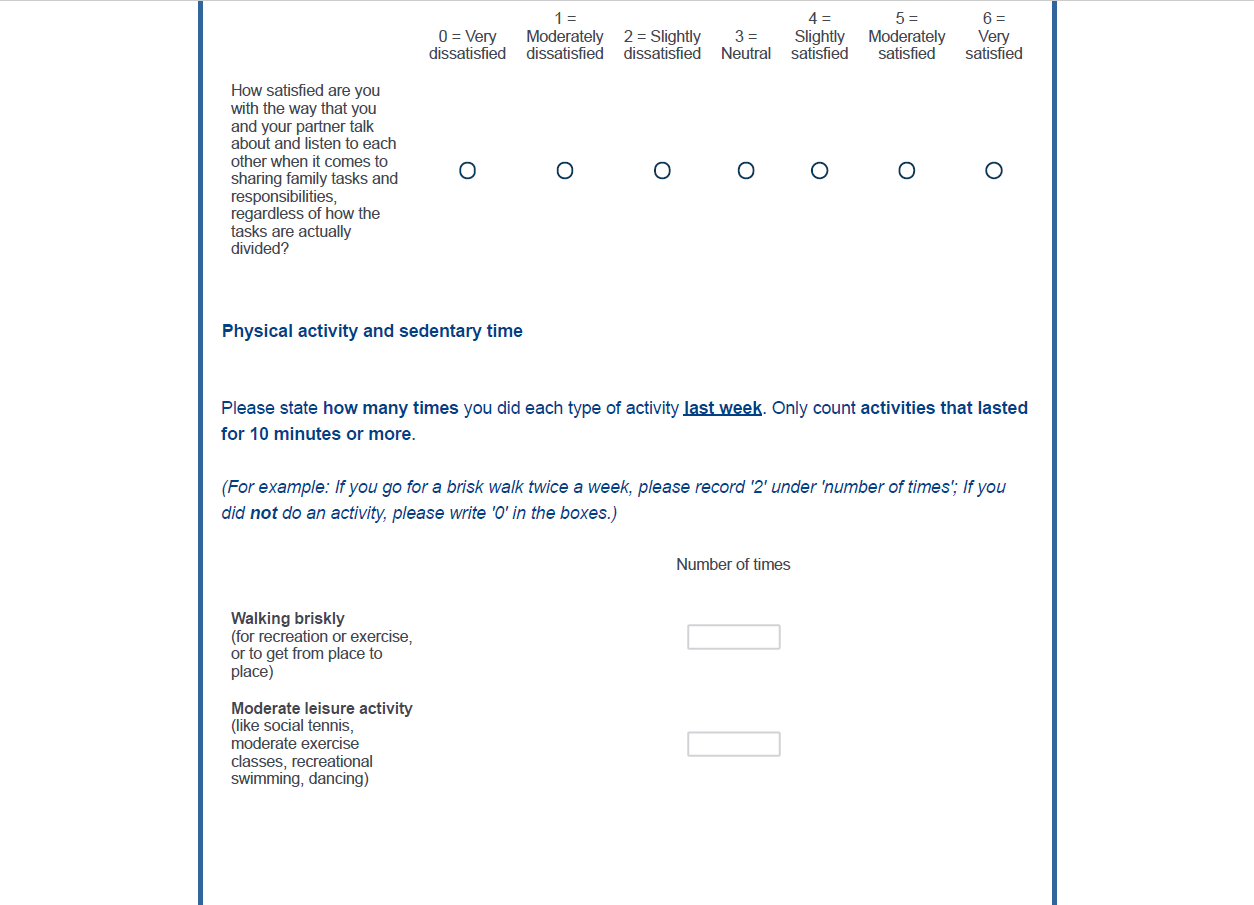


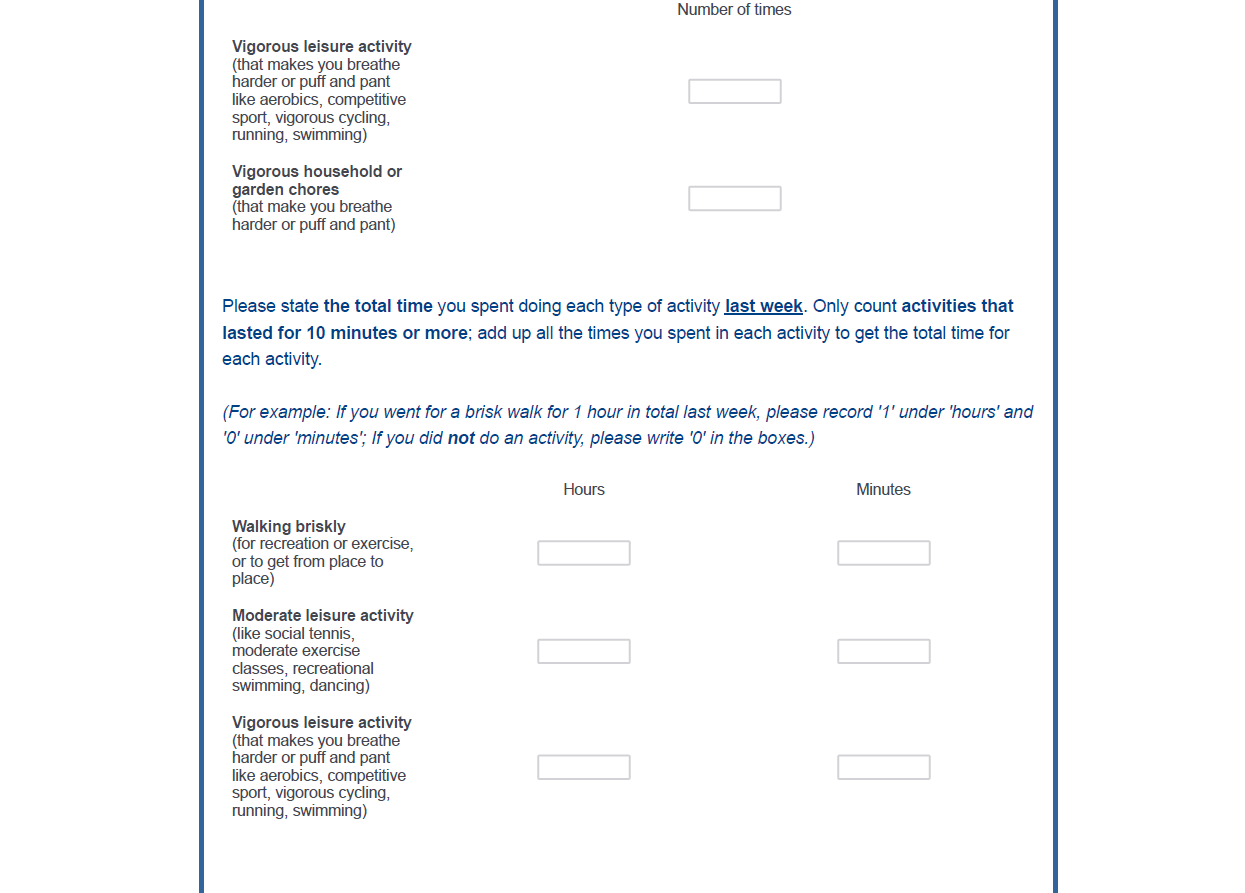


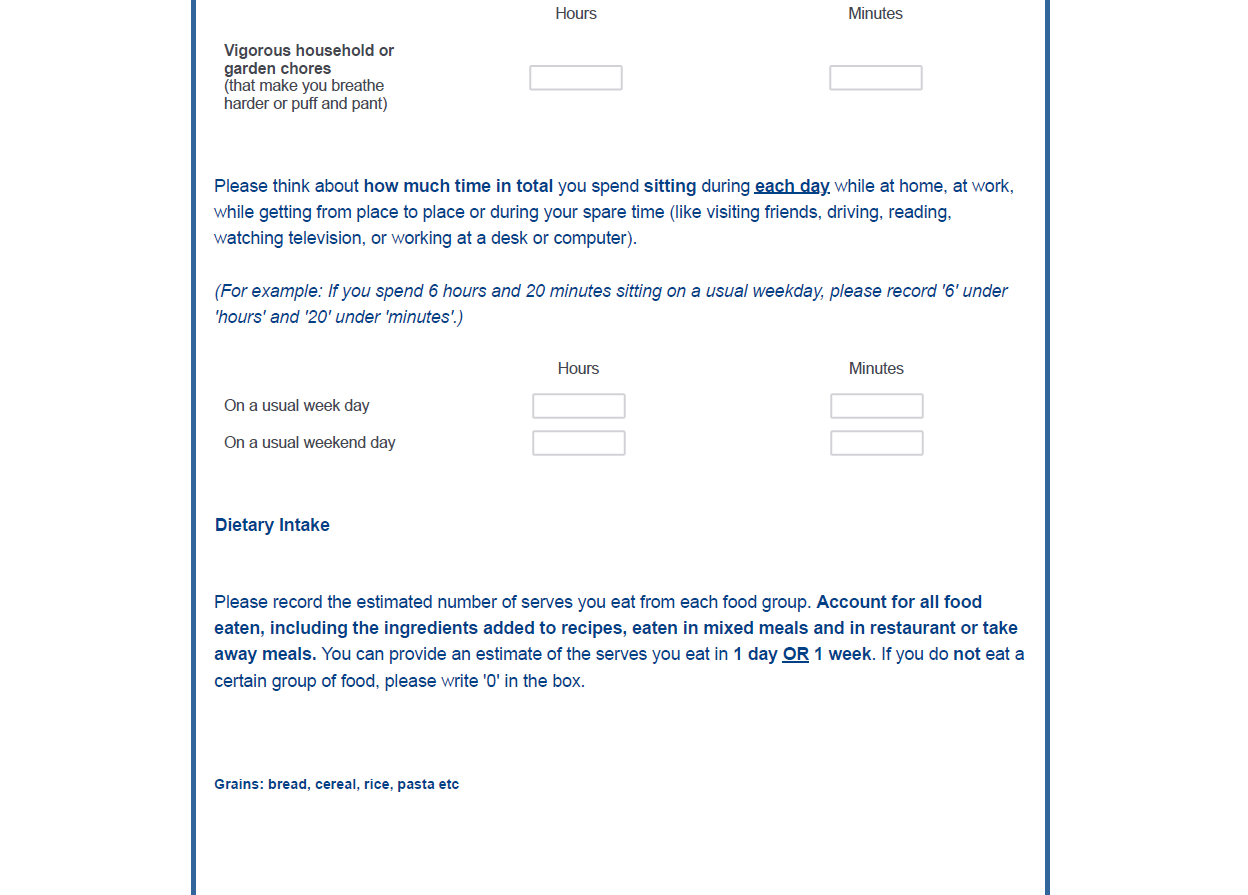


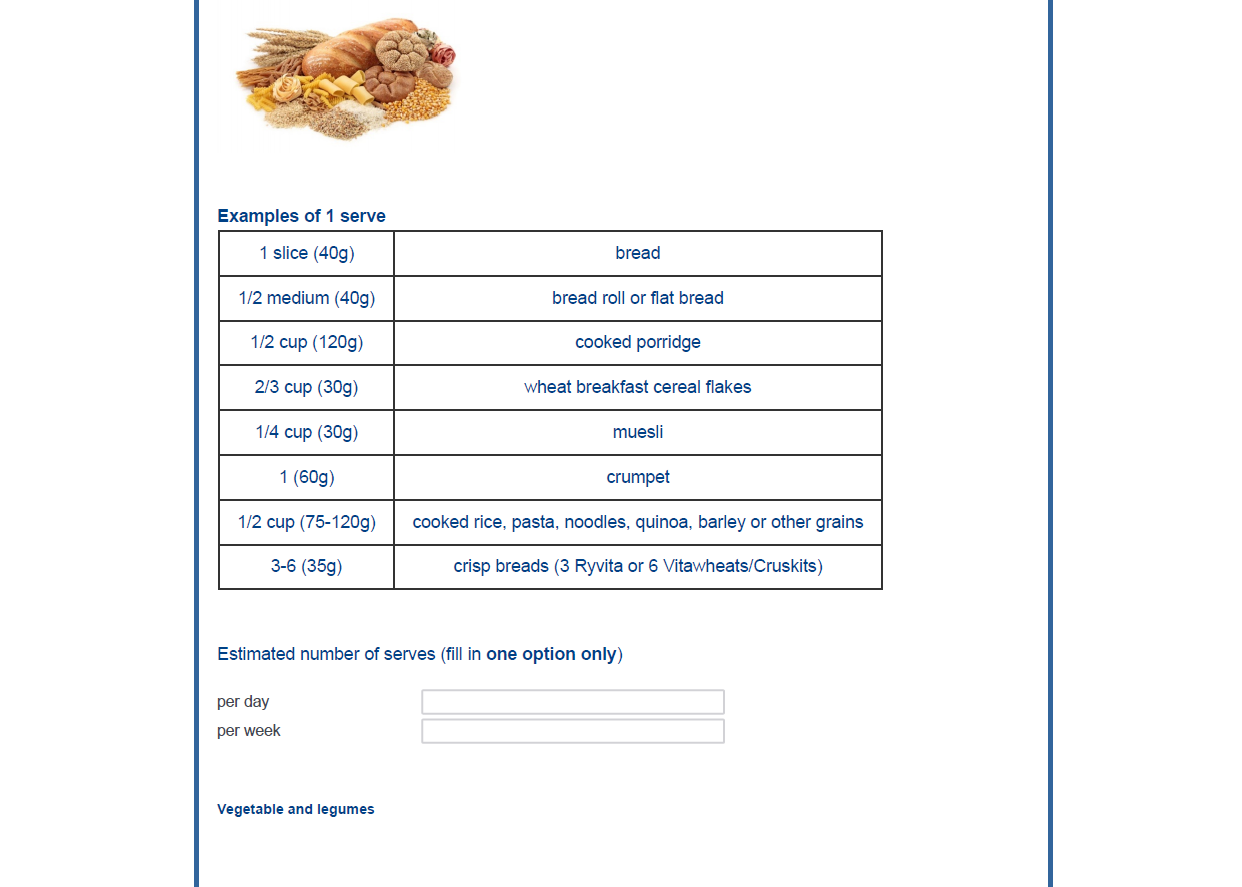


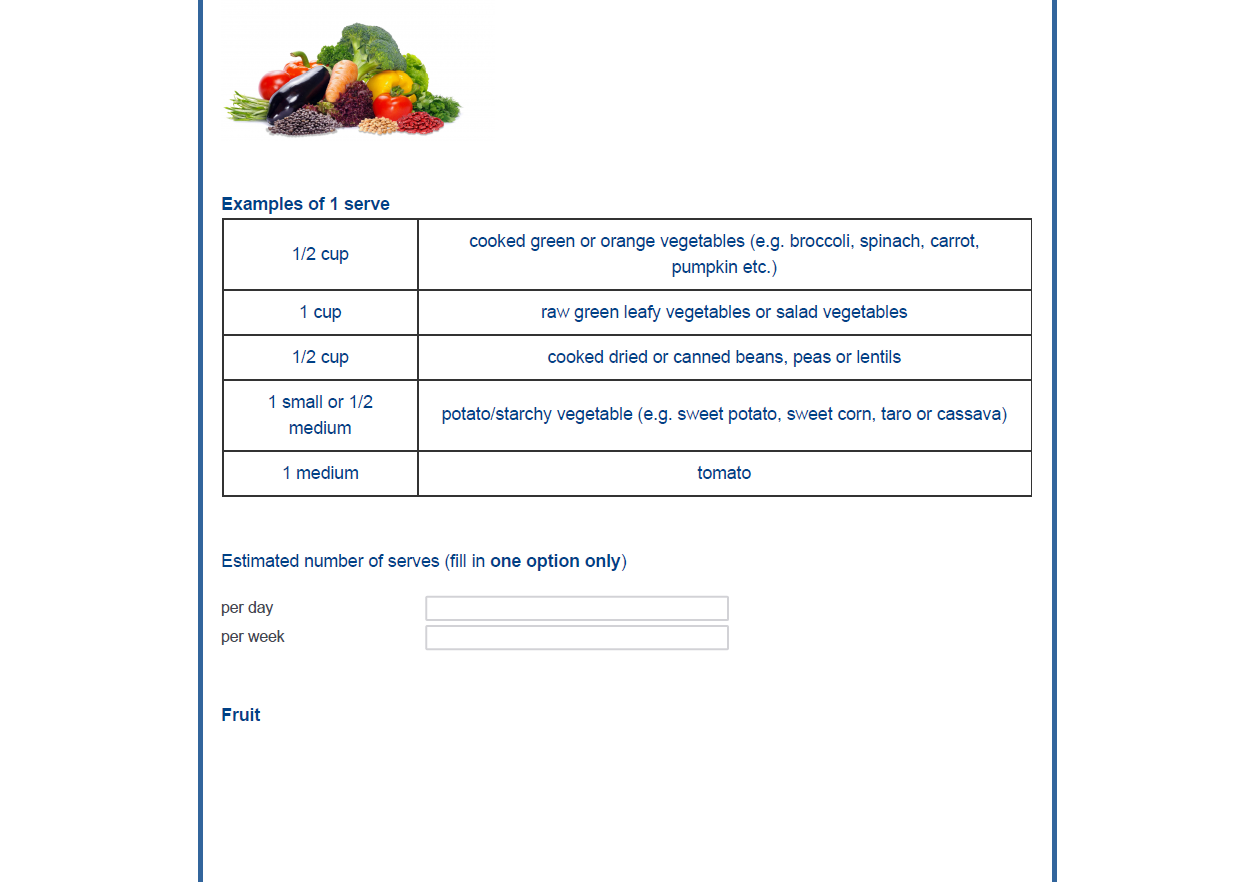


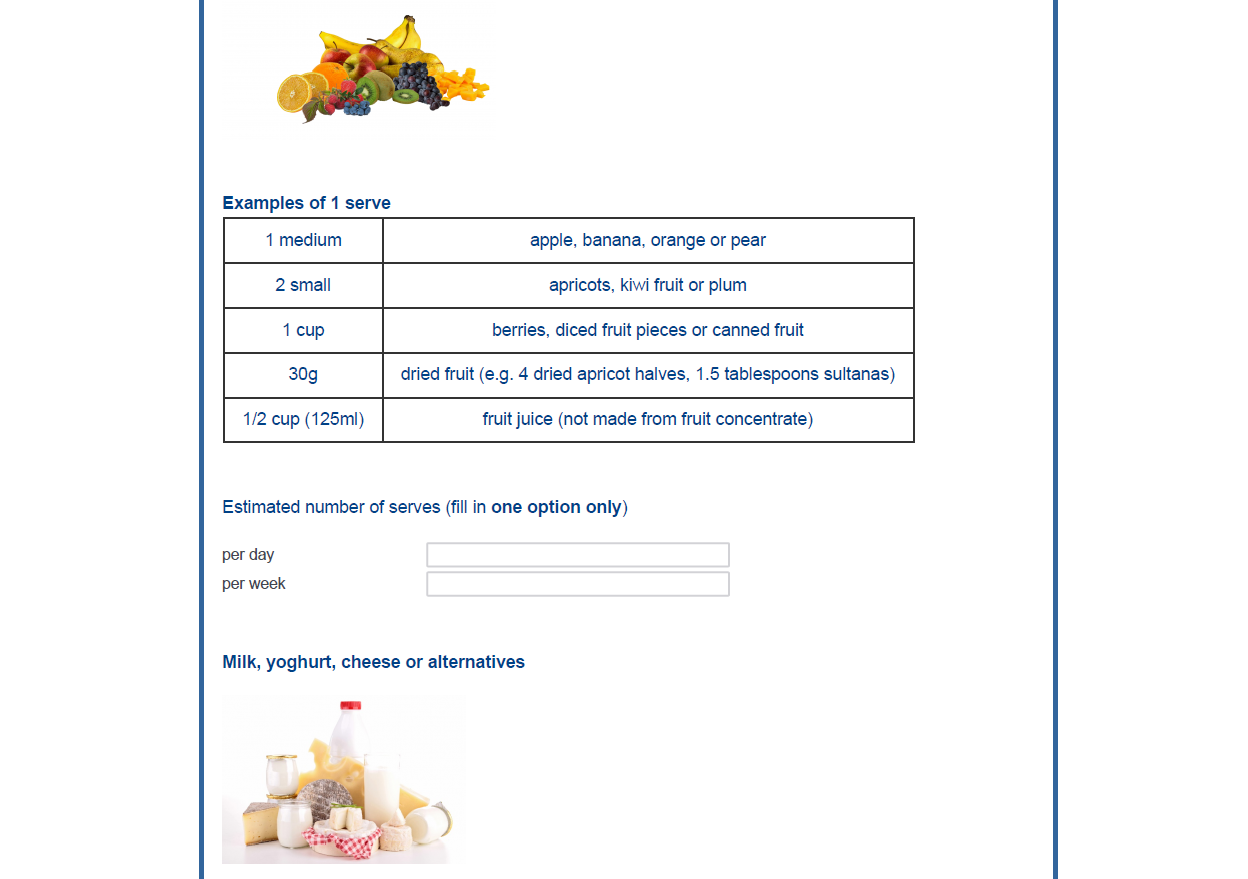


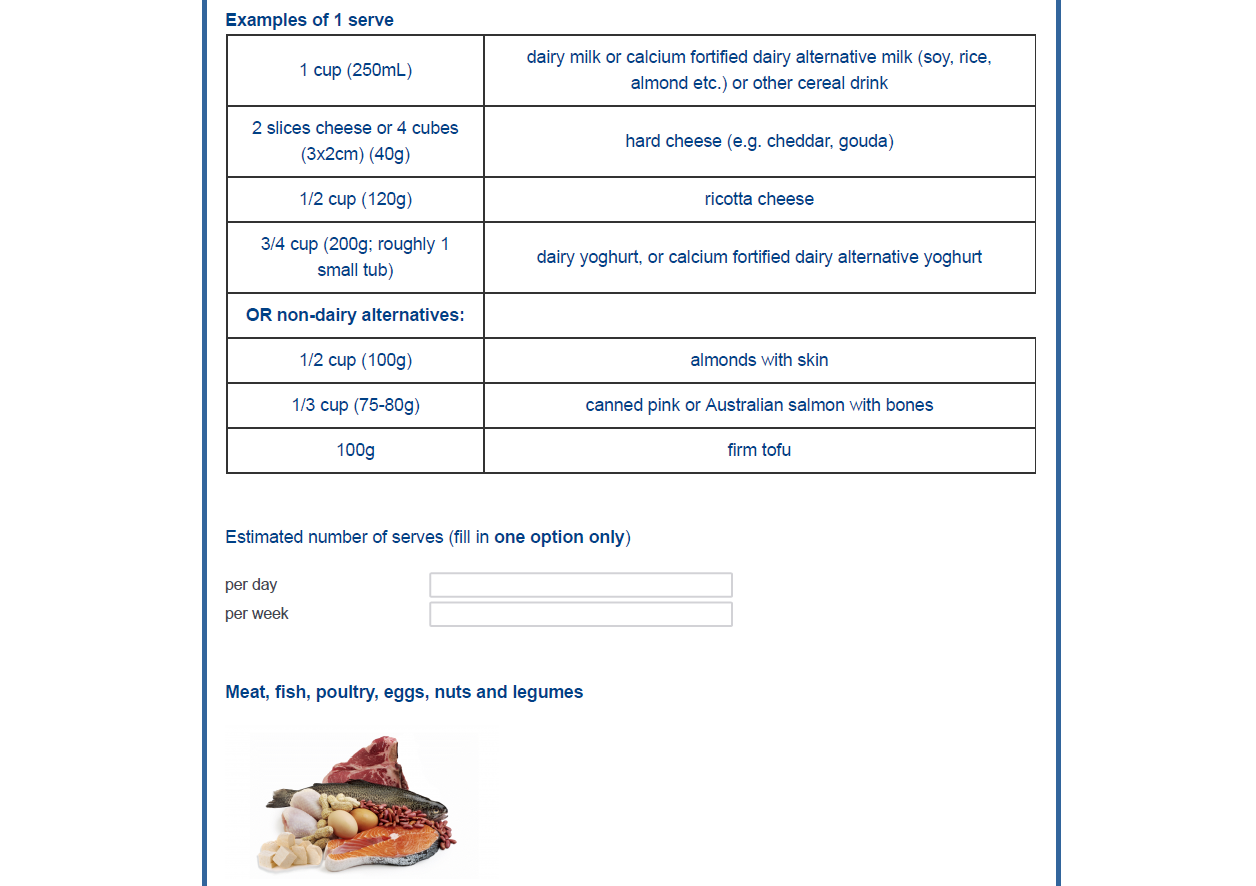


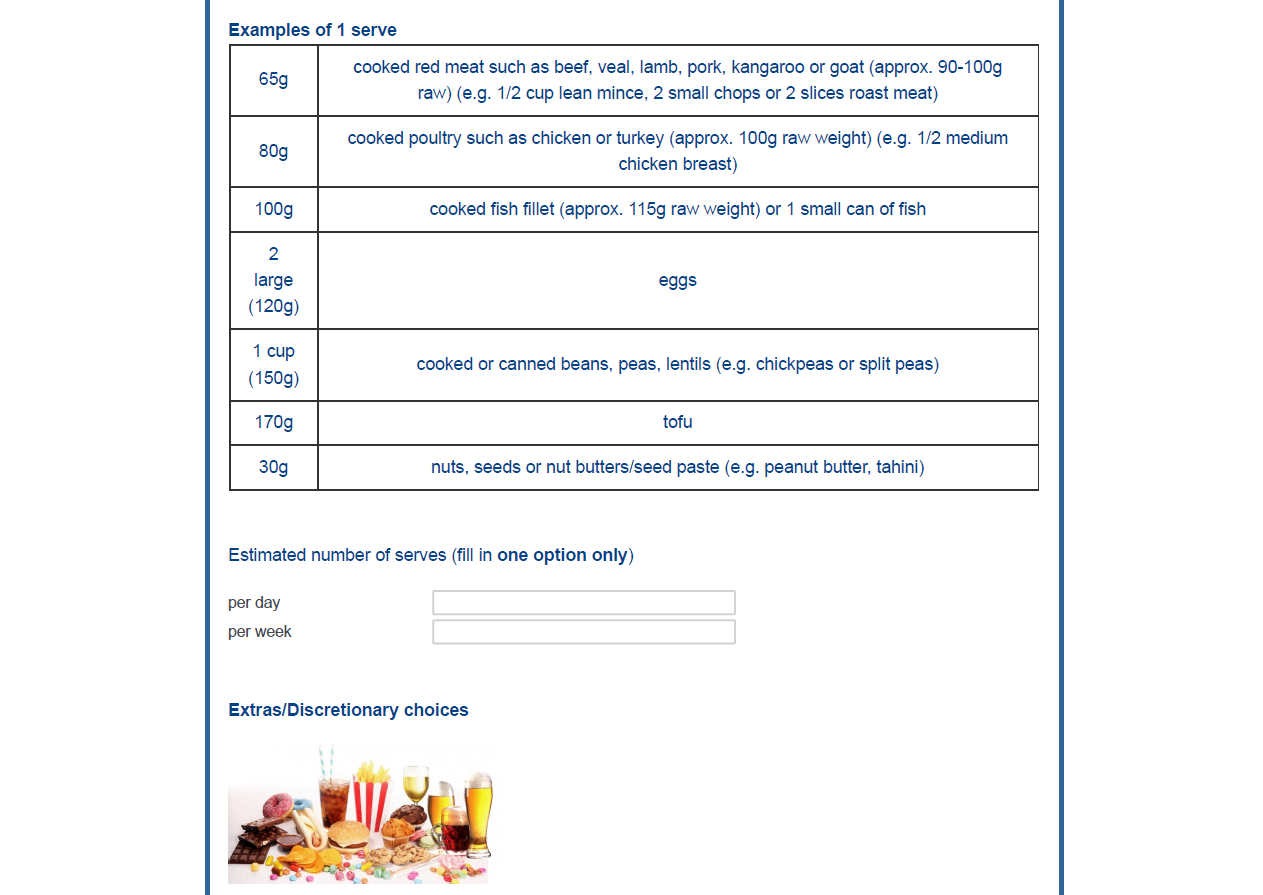


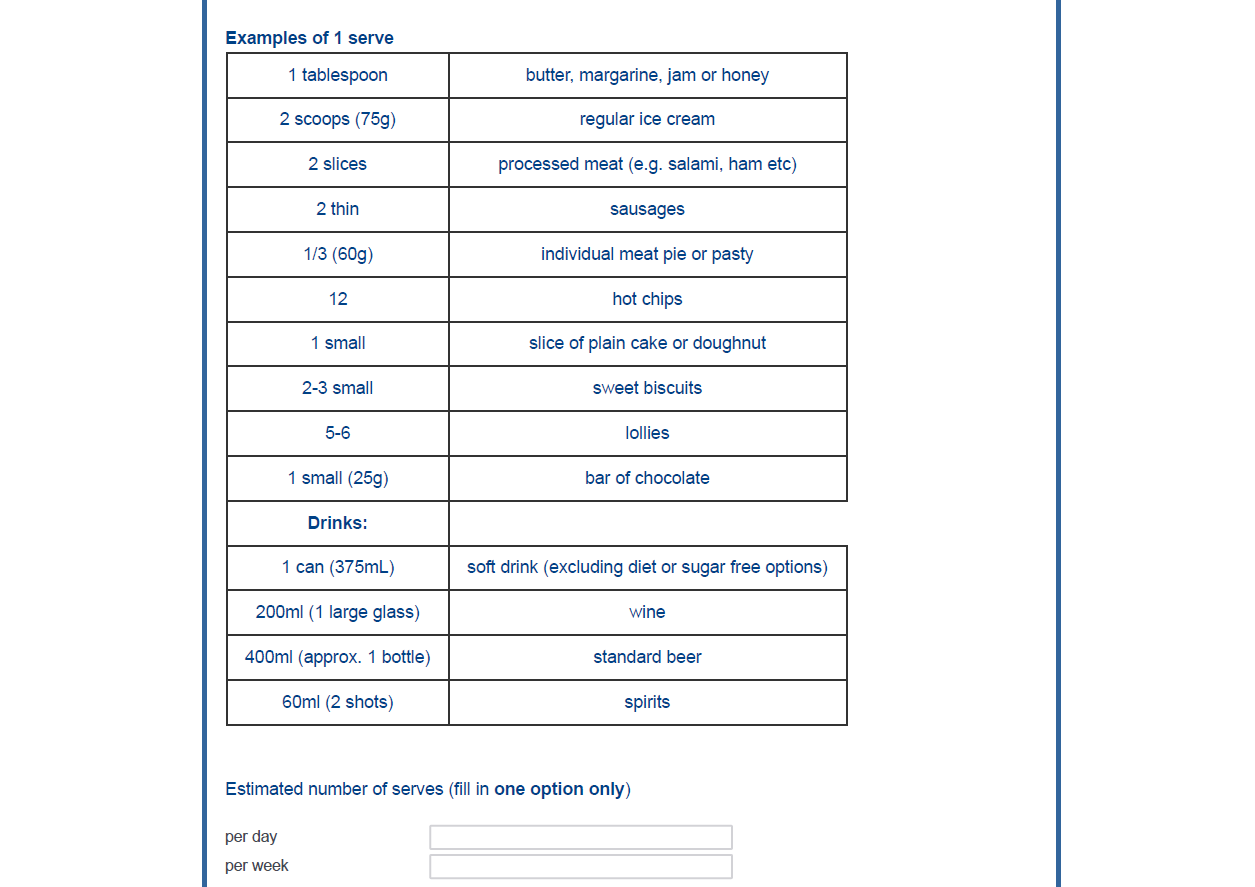


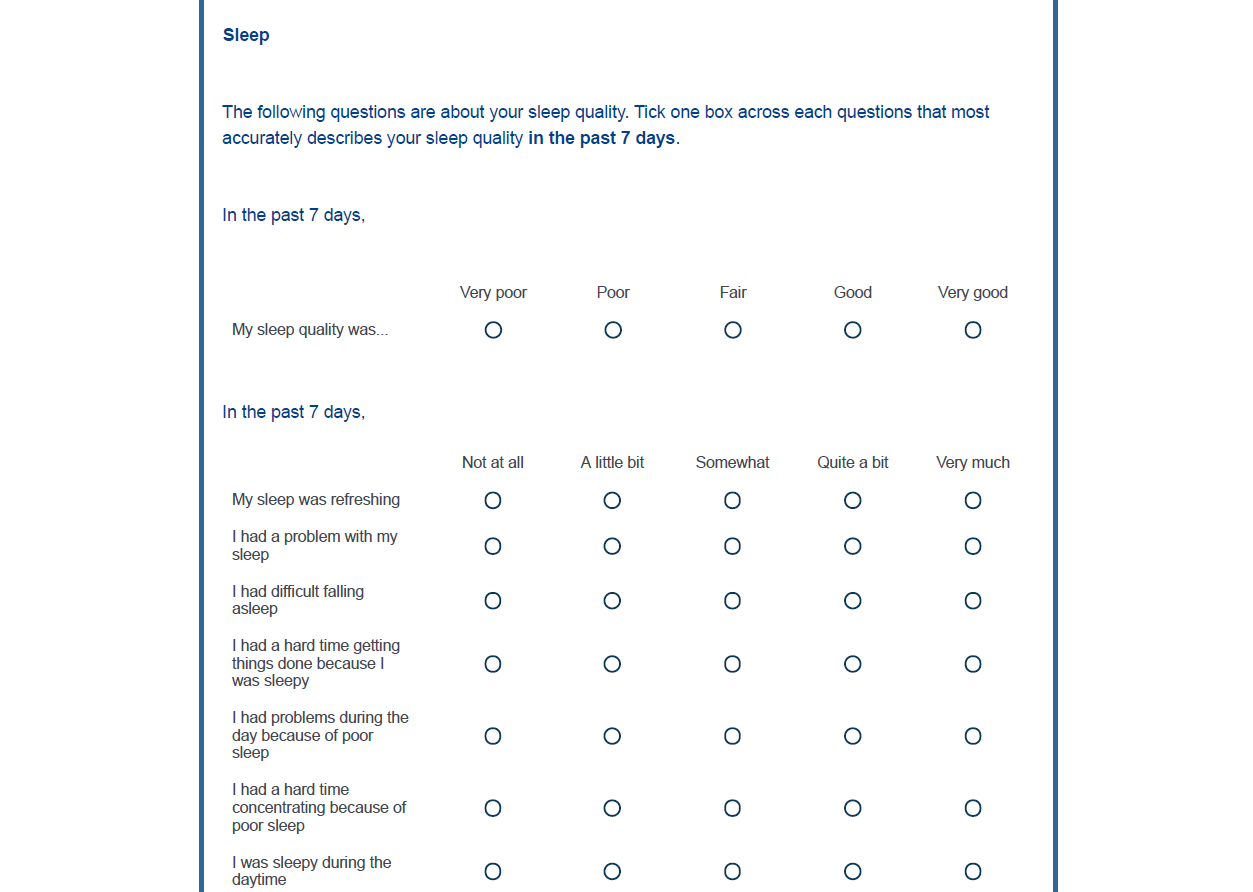


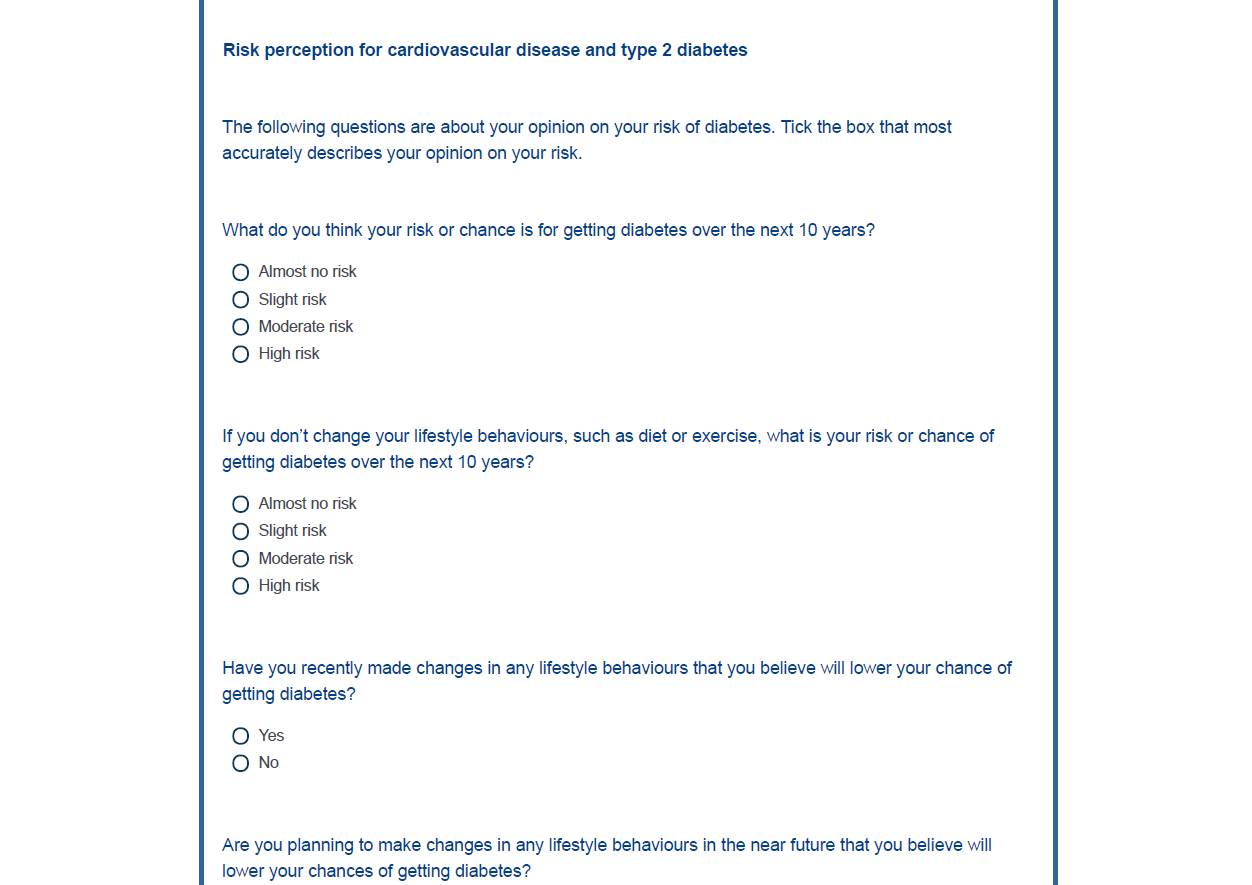


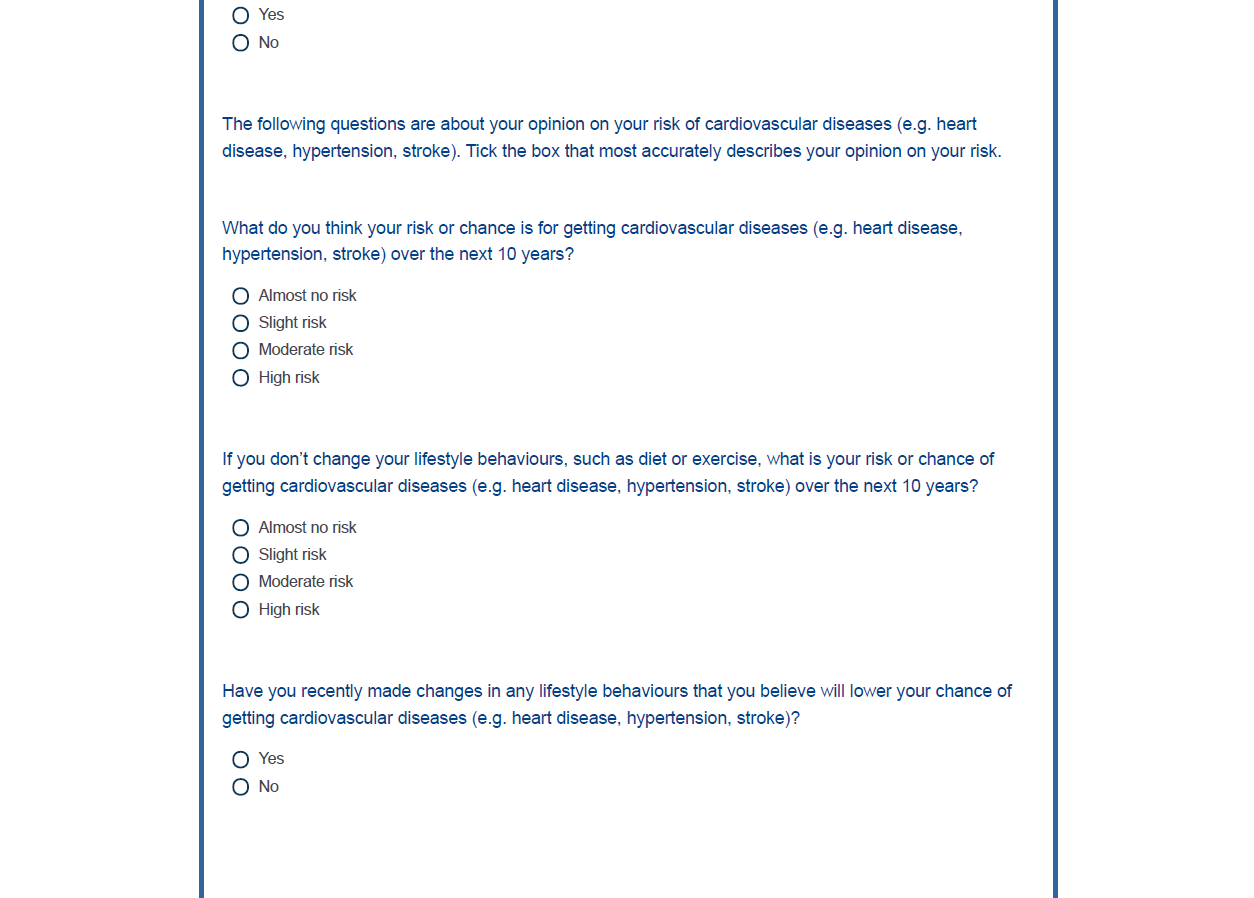


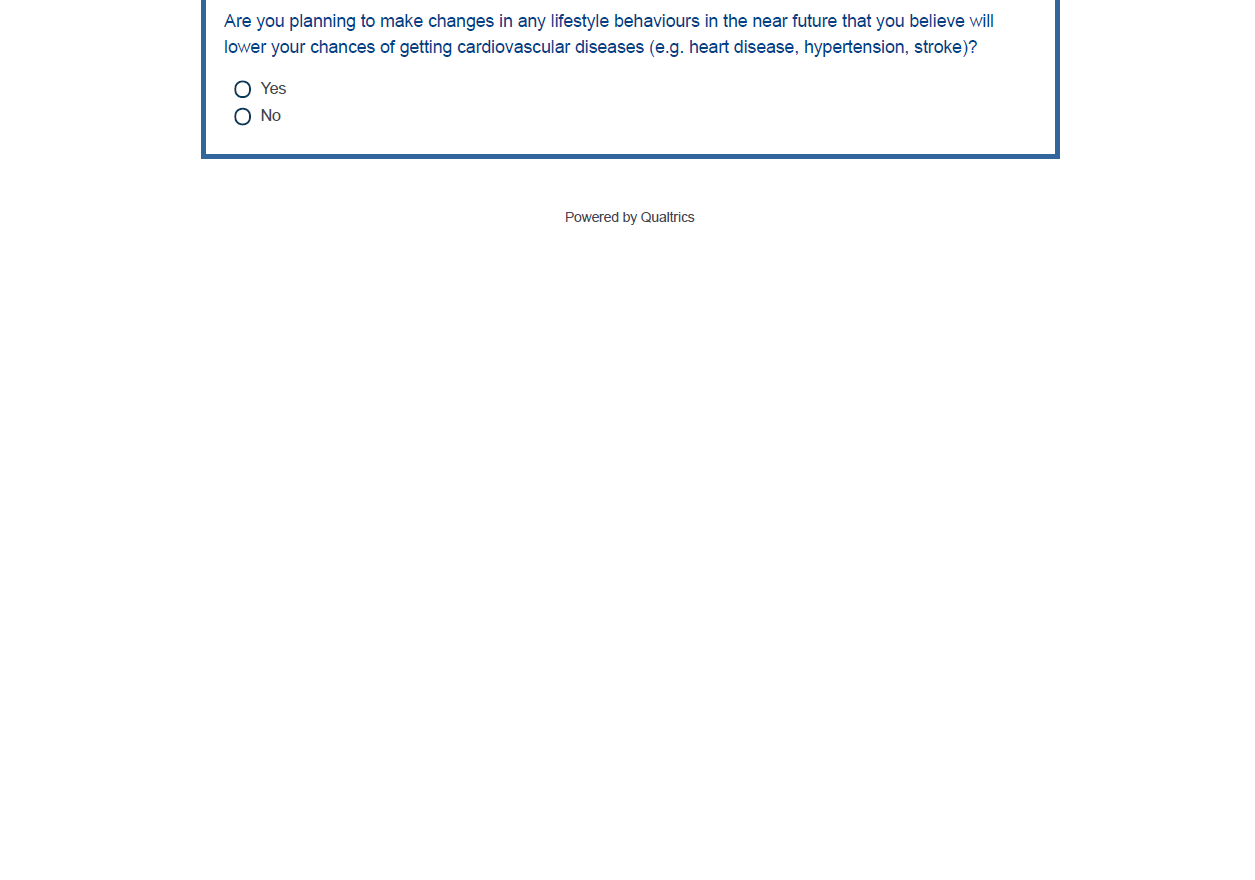

Supplement: sj-docx-1-whe-10.1177_17455057241247748 – Supplemental material for Preferred lifestyle intervention characteristics and behaviour change needs of postpartum women following cardiometabolic pregnancy complications [file sj-docx-1-whe-10.1177_17455057241247748.docx]
